# Patterns of genomic instability in > 2000 patients with ovarian cancer across six clinical trials evaluating olaparib

**DOI:** 10.1186/s13073-024-01413-5

**Published:** 2024-12-18

**Authors:** Alan Barnicle, Isabelle Ray-Coquard, Etienne Rouleau, Karen Cadoo, Fiona Simpkins, Carol Aghajanian, Alexandra Leary, Andrés Poveda, Stephanie Lheureux, Eric Pujade-Lauraine, Benoit You, Jonathan Ledermann, Ursula Matulonis, Charlie Gourley, Kirsten M. Timms, Zhongwu Lai, Darren R. Hodgson, Cathy E. Elks, Simon Dearden, Coumaran Egile, Pierre Lao-Sirieix, Elizabeth A. Harrington, Jessica S. Brown

**Affiliations:** 1 Translational Medicine, Oncology R&D, AstraZeneca, Cambridge Biomedical Campus, 1 Francis Crick Avenue, Cambridge, CB2 0AA UK; 2 Medical Oncology Department, Centre Léon Bérard and University Claude Bernard Lyon, and Groupe d’Investigateurs Nationaux pour l’Etude des Cancers Ovariens (GINECO), Lyon, France; 3 Department of Medical Biology and Pathology, Gustave Roussy, Cancer Genetics Laboratory, Gustave Roussy, Villejuif, France; 4 Memorial Sloan Kettering Cancer Center, New York, NY USA; 5 Department of Obstetrics and Gynecology, Jordan Center for Gynecologic Oncology at the Abramson Cancer Center, University of Pennsylvania, Philadelphia, PA USA; 6 Institut Gustave Roussy, and GINECO, Villejuif, France; 7 Initia Oncology, Hospital Quironsalud, Valencia, Spain; 8 Department of Medical Oncology, Princess Margaret Hospital, Toronto, ONT Canada; 9 Association de Recherche Cancers Gynécologiques (ARCAGY)-GINECO, and GINECO, Paris, France; 10 Medical Oncology, IC-HCL, EPSILYON, Univ Lyon 1, and GINECO, Lyon, France; 11 UCL Cancer Institute, University College London and UCL Hospitals, London, UK; 12 Dana-Farber Cancer Institute, Boston, MA USA; 13 Cancer Research UK Scotland Centre, University of Edinburgh, Edinburgh, UK; 14 Myriad Genetics, Salt Lake City, UT USA; 15 Translational Medicine, Oncology R&D, Research and Early Development, AstraZeneca, Boston, MA USA; 16 Precision Medicine and Biosamples, Oncology R&D, AstraZeneca, Cambridge, UK

**Keywords:** Ovarian cancer, Genomic instability, Translational research, Olaparib

## Abstract

**Background:**

The introduction of poly(ADP-ribose) polymerase (PARP) inhibitors represented a paradigm shift in the treatment of ovarian cancer. Genomic data from patients with high-grade ovarian cancer in six phase II/III trials involving the PARP inhibitor olaparib were analyzed to better understand patterns and potential causes of genomic instability.

**Patients and methods:**

Homologous recombination deficiency (HRD) was assessed in 2147 tumor samples from SOLO1, PAOLA-1, Study 19, SOLO2, OPINION, and LIGHT using next-generation sequencing technology. Genomic instability scores (GIS) were assessed in *BRCA1* and/or *BRCA2* (BRCA)*-*mutated (BRCAm), non-BRCA homologous recombination repair-mutated (non-BRCA HRRm), and non-HRRm tumors.

**Results:**

BRCAm was identified in 1021/2147 (47.6%) tumors. BRCAm tumors had significantly higher GIS than non-BRCAm tumors (*P* < 0.001) and high biallelic loss (815/838; 97.3%) regardless of germline (658/672; 97.9%) or somatic (101/108; 93.5%) BRCAm status. In non-BRCA HRRm tumors (*n* = 121) a similar proportion were HRD-positive (GIS ≥ 42: 55/121; 45.5%) relative to HRD-negative (GIS < 42: 52/121; 43.0%). GIS was highly variable in non-BRCA HRRm (median 42 [interquartile range (IQR) 29–58]) and non-HRRm (*n* = 1005; median 32 [IQR 20–55]) tumors. Gene mutations with high GIS included HRR genes *BRIP1* (median 46 [IQR 41–58]),* RAD51C* (median 58 [IQR 48–66]),* RAD51D* (median 62 [IQR 54–69]), and *PALB2* (median 64 [IQR 58–74]), and non-HRR genes *NF1* (median 49 [IQR 25–60]) and *RB1* (median 55 [IQR 30–71]). *CCNE1*-amplified and *PIK3CA*-mutated tumors had low GIS (*CCNE1*-amplified: median 24 [IQR 18–29]; *PIK3CA*-mutated: median 32 [IQR 14–52]) and were predominantly non-BRCAm.

**Conclusions:**

These analyses provide valuable insight into patterns of genomic instability and potential drivers of HRD, besides BRCAm, in ovarian cancer and will help guide future research into the potential clinical effectiveness of anti-cancer treatments in ovarian cancer, including PARP inhibitors as well as other precision oncology agents.

**Trial registration:**

The SOLO1 trial was registered at ClinicalTrials.gov (NCT01844986) on April 30, 2013; the PAOLA-1 trial was registered at ClinicalTrials.gov (NCT02477644) on June 18, 2015 (retrospectively registered); Study 19 was registered at ClinicalTrials.gov (NCT00753545) on September 12, 2008 (retrospectively registered); the SOLO2 trial was registered at ClinicalTrials.gov (NCT01874353) on June 7, 2013; the OPINION trial was registered at ClinicalTrials.gov (NCT03402841) on January 3, 2018; the LIGHT trial was registered at ClinicalTrials.gov (NCT02983799) on November 4, 2016.

**Supplementary Information:**

The online version contains supplementary material available at 10.1186/s13073-024-01413-5.

## Background

Homologous recombination repair (HRR) is a DNA repair pathway that acts on double-strand breaks and interstrand cross-links [1]. A deficiency in the HRR pathway—known as homologous recombination deficiency (HRD)—is associated with various tumor types, most prominently ovarian, breast, prostate, and pancreatic cancers [1]. The HRD phenotype is associated with various signatures of genomic instability [1], including loss of heterozygosity (LOH) [2], telomeric allelic imbalances (TAIs) [3], and large-scale state transitions (LSTs) [4]. The identification of homologous recombination deficient tumors can be achieved either by measuring these signatures of genomic instability (HRD testing) or by detecting mutations in the breast cancer susceptibility genes *BRCA1* and/or *BRCA2* (BRCAm) as well as by detecting other HRR pathway defects [1]*.*

Poly(ADP-ribose) polymerase (PARP) inhibitors are a class of anti-cancer drugs that target tumors with HRD. The mechanism of action involves trapping of PARP at sites of single-stranded DNA breaks, resulting in replication-dependent DNA double-strand breaks that cannot be repaired accurately in HRD-positive tumors [5, 6]. Three different PARP inhibitors have received global regulatory approval as first-, second-, or later-line therapy in ovarian cancer, and some of these are also approved therapies in breast, prostate, and pancreatic cancers (see Additional file 1 for further details).

High-grade serous ovarian cancer was considered a promising tumor type for PARP inhibitor therapy as it is characterized by a high frequency of genetic alterations in *BRCA1* and/or *BRCA2* and genomic instability [7]. In newly diagnosed patients with ovarian cancer, the benefit from maintenance PARP inhibitors has been greatest in patients with a BRCAm or HRD-positive tumors [8–11]. However, the drivers of HRD in ovarian cancer, other than mutations in *BRCA1* and *BRCA2,* are not well understood.

We analyzed genomic data from six olaparib global phase II/III studies (Additional file 1: Table S1) [8, 9, 12–22]. These included over 2000 patients with ovarian cancer in whom molecular profiling including genomic instability was assessed. Our objective was to describe patterns of genomic instability in BRCAm and non-BRCAm tumors and better understand drivers of HRD in the newly diagnosed and platinum-sensitive relapsed ovarian cancer (PSROC) populations.

## Methods

### Study design, participants, and tumor samples

This was a pooled analysis of data from the SOLO1 [8, 13], PAOLA-1 [9, 22], Study 19 [14–16], SOLO2 [18, 21], OPINION [19, 20], and LIGHT [12, 17] clinical trials of olaparib in high-grade serous or high-grade endometrioid ovarian cancer. Details of these studies have been reported previously and are summarized in Additional file 1: Table S1. All trials and analyses were performed in accordance with the principles of the Declaration of Helsinki and Good Clinical Practice guidelines and were approved by the appropriate Institutional Review Boards. All patients provided written informed consent.

Archival tumor samples, predominantly obtained at diagnosis, from each study were used, and genomic DNA derived from formalin-fixed paraffin-embedded tumor tissue or whole blood samples was analyzed (Additional file 1: Table S2).

### Definitions and assays

Genomic DNA was used to assess tumor BRCAm (tBRCAm) status. Somatic BRCAm was defined as a positive tBRCAm status and negative germline BRCAm status; samples without a germline BRCAm status were classified as somatic/germline BRCAm status not determined. Genomic instability and *BRCA1*/*BRCA2* mutation status were measured using a version of the Myriad MyChoice® CDx next-generation sequencing-based tumor test developed by Myriad Genetics (Myriad Genetic Laboratories, Inc., Salt Lake City, UT, USA) (see Additional file 1: Table S2 for further details of the assays used).

Genomic instability was determined by measuring LOH (number of LOH regions longer than 15 Mb but shorter than the whole chromosome), TAI (number of regions with allelic imbalance that extend to one of the subtelomeres but do not cross the centromere and are longer than 11 Mb), and LSTs (number of break points between regions longer than 10 Mb after filtering out regions shorter than 3 Mb). LOH, TAI, and LST scores were combined to provide a total genomic instability score (GIS) of 0–100, with higher scores indicating greater genomic instability.

A tumor was determined to be HRD-positive if it had a tBRCAm or met the previously established GIS cutoff of ≥ 42 [23]; 42 was used as the GIS cutoff for the MyChoice® HRD Plus assay (now known as the MyChoice® CDx assay) in the PAOLA-1 [9], OPINION [19], and LIGHT [12] studies. A tumor was determined to be HRD-negative if the GIS was < 42 with non-tBRCAm status. HRD status was unknown for cases where the GIS could not be determined due to assay failure and the tumor was non-tBRCAm status. For example, samples that were low tumor purity were failed to ensure that false negative results were not reported.

Genomic instability was assessed in samples with *BRCA1*m, *BRCA2*m, and non-BRCA HRR mutations (non-BRCA HRRm), defined as loss-of-function mutations in 13 other genes involved in HRR: *ATM*, *BARD1*, *BRIP1*, *CDK12*, *CHEK1*, *CHEK2*, *FANCL*, *PALB2*, *PPP2R2A*, *RAD51B*, *RAD51C*, *RAD51D*, and *RAD54L*; *PPP2R2A* was included in the non-BRCA HRRm analysis because it was considered a non-BRCA HRRm gene in prespecified analyses of the included studies. Genomic instability was also assessed in samples with deleterious or suspected deleterious mutations other than BRCAm or non-BRCAm HRRm, including *MAP3K1*, *MYC*, *RB1*, *KMT2D*, *CSMD3*, *SOX2*, *NF1*, *MAP2K4*, *NTHL1*, *PTEN*, *STAG2*, *PTPRD*, *TP53*, *FANCM*, *ERBB2*, *TSC1*, *MCL1*, *BLM*, *PIK3CA*, *MYH*, *NBN*, *CCNE1*, *ARID1A*, *NRAS*, and *KRAS*, as determined by the Myriad tumor tissue gene panel (108 in total; full gene list is provided in Additional file 1: Table S3) and a Foundation Medicine gene panel (for Study 19 only).

Gene-specific LOH was defined by Myriad Genetics as LOH localized to a given locus and included biallelic inactivation (locus contains at least one homozygous mutation or two or more deleterious mutations in a single gene; no wild-type allele detected), heterozygous (locus contains a single deleterious mutation or alteration in one allele; one functional copy remains), and unknown (locus could not be determined as biallelic or heterozygous). Biallelic loss was initially determined by the LOH status of the chromosomal region encompassing a gene. The LOH status was unknown if a boundary between two regions, one with LOH (classified as “biallelic loss”) and one without LOH (classified as “heterozygous”), was located inside the gene. In addition, the initial LOH status was unknown if the GIS analysis failed. Initial LOH calls were manually reviewed and corrected (e.g., a patient carrying a deleterious or suspected deleterious BRCAm with LOH in *BRCA1*/*BRCA2* was considered to have a biallelic loss of *BRCA1*/*BRCA2*).

Tumors with co-occurring *BRCA1* and *BRCA2* alterations or with co-occurring individual non-BRCA HRR alterations were excluded from individual gene analyses as gene-specific HRD and zygosity on the patient level could not be assessed. Tumors with a non-HRR alteration were reported as having that gene alteration even if there was a co-occurring non-HRR alteration; however, tumors with a non-HRR alteration and a co-occurring *BRCA1* and/or *BRCA2* alteration or non-BRCA HRR alteration were considered BRCAm and non-BRCA HRRm, respectively.

### Statistical analyses

Statistical variance between groups was determined by applying an independent Student’s *t*-test, with a threshold of *P* < 0.05 regarded as statistically significant.

## Results

### Patterns of genomic instability in the analysis cohort

Across the six studies, sequencing results were available for tumors from 2147 patients, with a valid GIS available for 1838 tumors. Where GIS was evaluable, the median GIS observed across studies was 54.0 (interquartile range [IQR] 31–66). Patterns of GIS were assessed for different baseline characteristics of interest. The observed range of GIS did not differ considerably by patient race (White, Black or African American, Asian, other/unknown), across tumor histologies (serous, endometrioid, other/unknown), or by primary tumor location (ovary, fallopian tube, peritoneal, other/unknown) (Additional file 1: Fig S1) where evaluable. However, in this analysis cohort, the sample size was limited beyond patients with White race or tumors with serous histology.

### GIS in BRCAm tumors

**Fig. 1:**
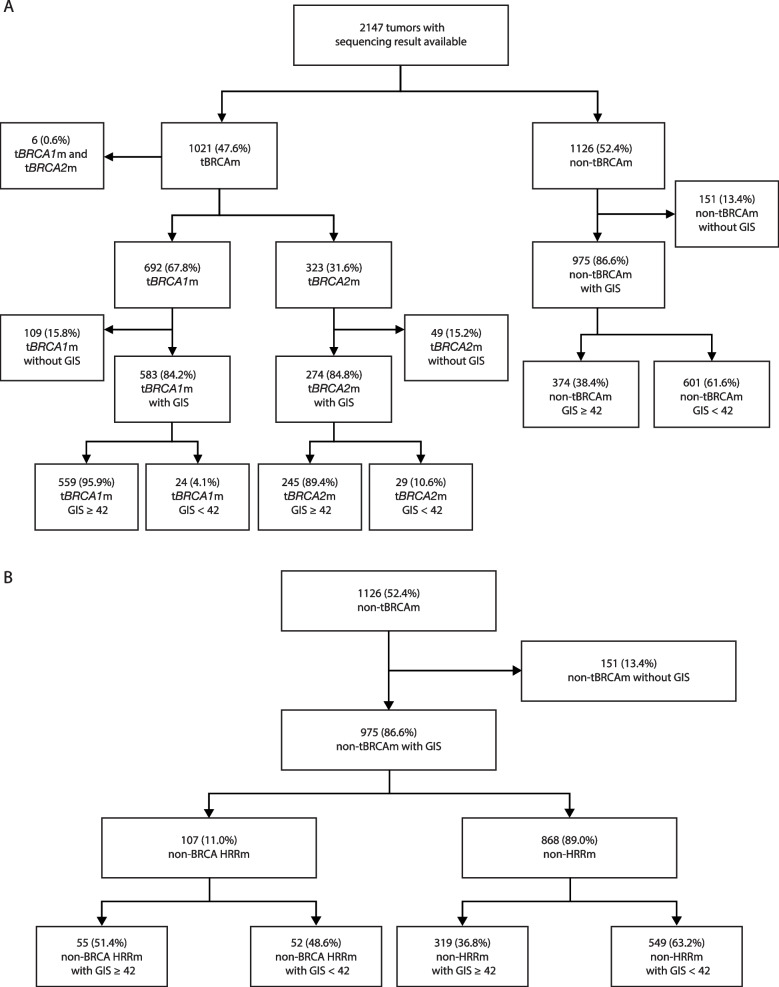
GIS status in **A** t*BRCA1*m and t*BRCA2*m and non-tBRCAm and **B** non-BRCA HRRm and non-HRRm. *BRCA1*m, *BRCA1* mutation; *BRCA2*m, *BRCA2* mutation; GIS, genomic instability score; HRRm, homologous recombination repair mutation; t, tumor

**Fig. 2:**
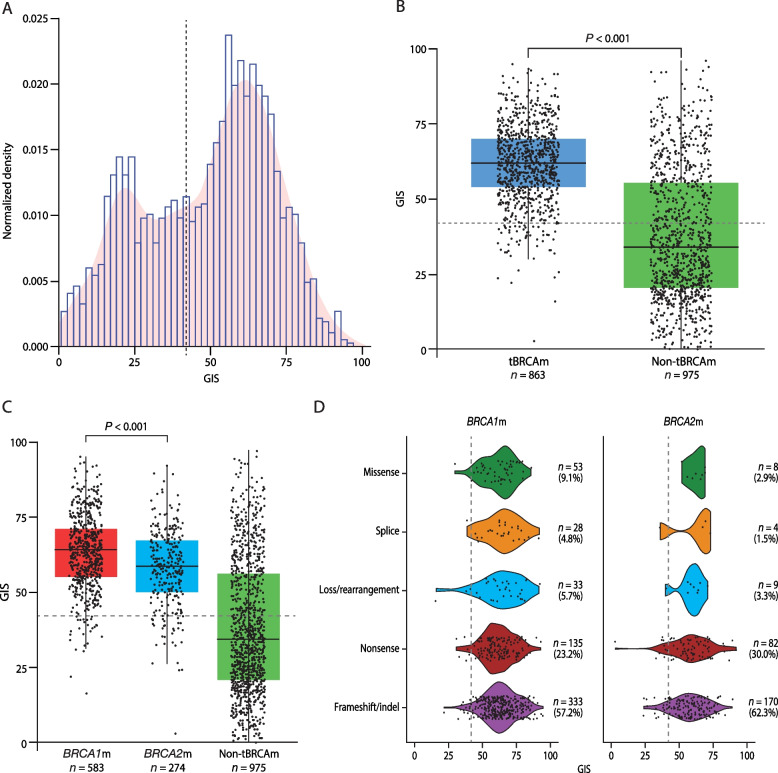
GIS distribution **A** overall, **B** in tumors with and without tBRCAm, **C** in tumors with *BRCA1*m and *BRCA2*m and without tBRCAm, and **D** in *BRCA1*m and *BRCA2*m mutation subtypes. In panel **A**, distributions were based on 1838 tumors with measurable GIS (623 samples from PAOLA-1, 241 from OPINON, 344 from LIGHT, 226 from SOLO1, 210 from SOLO2, and 194 from Study 19). The dashed vertical line denotes the GIS cutoff of 42. In panels **B** and **C**, the box plot shows median (IQR) and whiskers indicate 1.5 times the IQR above Q3 and below Q1. The dashed horizontal line denotes the GIS cutoff of 42. Panel **C** excludes tumors with co-occurring *BRCA1* and *BRCA2* mutations. In panel **D**, 582 *BRCA1*m tumors and 273 *BRCA2*m tumors were evaluable. Excludes patients with *BRCA1* or *BRCA2* double-hit mutations (*n* = 2) and co-occurring HRRm and non-BRCAm genes (*n* = 4). The dashed vertical lines denote the GIS cutoff of 42. *BRCA1*m, *BRCA1* mutation; *BRCA2*m, *BRCA2* mutation; BRCAm, *BRCA1* and/or *BRCA2* mutation; GIS, genomic instability score; HRRm, homologous recombination repair mutation; IQR, interquartile range; Q, quartile; tBRCAm, tumor BRCAm

Distinct patterns of GIS were observed by mutation status. Of the 2147 tumors with available sequencing results, 1021 (47.6%) had tBRCAm, including 692 (67.8%) with t*BRCA1*m, 323 (31.6%) with t*BRCA2*m, and 6 (0.6%) with both t*BRCA1*m and t*BRCA2*m; 1126 (52.4%) were non-tBRCAm (Fig. 1). The majority of tumors with tBRCAm had a valid GIS (*n* = 863 [84.5%]; *n* = 583 [84.2%] with t*BRCA1*m alone and *n* = 274 [84.8%] with t*BRCA2*m alone), and most had a GIS ≥ 42 (*n* = 810 [93.9%]; *n* = 559 [95.9%] with t*BRCA1*m alone and *n* = 245 [89.4%] with t*BRCA2*m alone) (Fig. 1). Where GIS was measurable in the overall study population (*n* = 1838), the distribution of GIS was bimodal (Fig. 2A). Those with tBRCAm predominantly had high GIS (median 62 [IQR 54–70]) and tBRCAm tumors had a significantly higher median GIS than non-tBRCAm tumors (median 34 [IQR 21–56]; *P* < 0.001) (Fig. 2B). This pattern was observed for both *BRCA1*m and *BRCA2*m patients; however, a significantly higher GIS was seen in *BRCA1*m (median GIS 64 [IQR 55–71]) than *BRCA2*m (median GIS 59 [IQR 50–67]) tumors (*P* < 0.001; Fig. 2C).

The higher GIS for both *BRCA1*m and *BRCA2*m (relative to non-tBRCAm) was consistent across the individual studies (Additional file 1: Fig. S2) and in patients with newly diagnosed ovarian cancer (Additional file 1: Fig. S3A) or PSROC (Additional file 1: Fig. S3B). GIS patterns were similar regardless of mutation subtype, including missense, splice, loss/rearrangement, nonsense, and frameshift/indel alterations (Fig. 2D).

As detailed in the Methods section, the origin of BRCAm (germline or somatic) was assessed by germline testing of blood or by computational assessment of germline/somatic status of tBRCAm in cases where germline testing was not conducted. Across all studies, > 95% of germline BRCAm reported by blood testing were also reported based on tumor tissue testing (Additional file 1: Table S2). Of t*BRCA1*m or t*BRCA2*m samples with a valid GIS (*n* = 857), 693 (80.9%) were germline BRCAm, 113 (13.2%) were somatic BRCAm, and 51 (6.0%) could not be classified as germline or somatic and their origin were considered as undetermined. Median GIS was similar regardless of whether *BRCA1*m and *BRCA2*m were of germline origin (germline *BRCA1*m: median 64 [IQR: 56–71]; germline *BRCA2*m: median 59 [IQR 50–68]), somatic origin (somatic *BRCA1*m: median 62 [IQR: 56–71]; somatic *BRCA2*m: median 57 [IQR 44–65]), or undetermined origin (undetermined *BRCA1*m: median 63 [IQR 54–70]; undetermined *BRCA2*m: median 60 [IQR 50–67]) (Additional file 1: Fig. S3C). Gene-specific LOH (biallelic inactivation) was observed in almost all evaluable BRCAm tumors (815/838; 97.3%): 572/574 (99.7%) of those with t*BRCA1*m and 243/264 (92.0%) with t*BRCA2*m; monoallelic loss was seen in the remaining evaluable tumors (Additional file 1: Fig. S4A). The higher GIS seen in *BRCA1* tumors may be partially explained by the slightly lower rate of monoallelic loss observed in *BRCA1* than in *BRCA2*. When assessing gene-specific LOH and GIS patterns together in BRCAm tumors (*n* = 825), the GIS was high in tumors with biallelic inactivation (median GIS 62 [IQR 55–70]), which accounted for the majority of BRCAm (802/825, 97.2%) (Additional file 1: Fig. S4B). GIS was relatively lower in heterozygous BRCAm tumors (median GIS 39; IQR 33–53), which accounted for the minority of BRCAm (23/825, 2.8%) (Additional file 1: Fig. S4B). Evaluation of gene-specific LOH revealed that the rate of biallelic inactivation was high for tumors irrespective of the origin, with a germline (658/672; 97.9%) or somatic (101/108; 93.5%) BRCAm (Additional file 1: Fig. S4C). Gene-specific zygosity in patients with *BRCA1*m or *BRCA2*m by individual study is shown in Additional file 1: Fig. S5.

### GIS in non-BRCA HRRm tumors

**Fig. 3:**
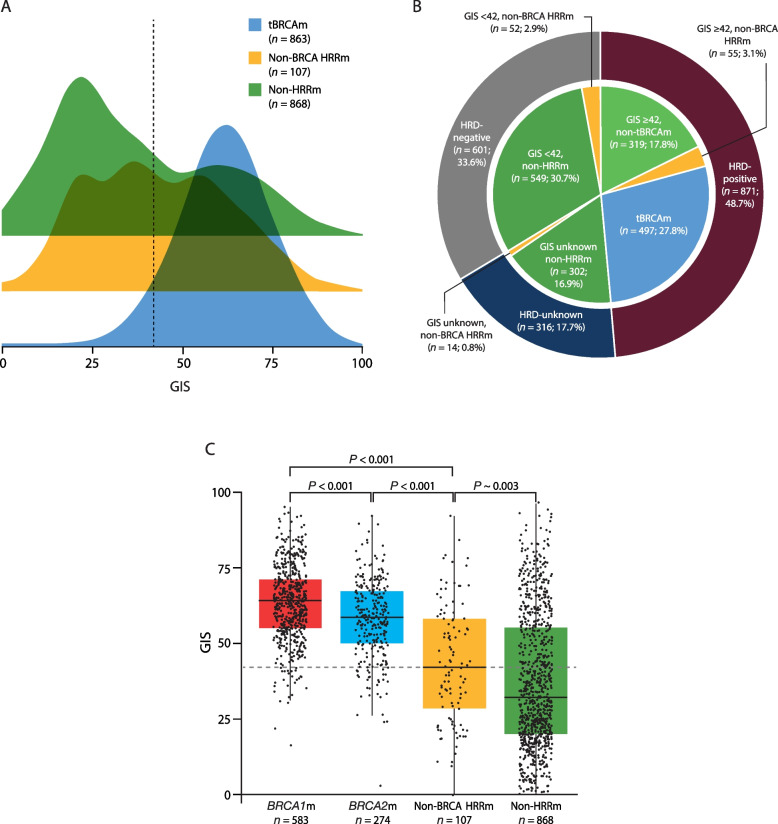
GIS distribution **A** in tumors with tBRCAm, non-BRCA HRRm, and non-HRRm, **B** relative proportion of tBRCAm, non-BRCA HRRm, and non-HRRm status in the context of HRD-positive, HRD-negative, and HRD-unknown biomarker status in PAOLA-1, OPINION, LIGHT, and Study 19, and **C** GIS distribution in tumors with *BRCA1*m, *BRCA2*m, non-BRCA HRRm, and non-HRRm biomarker status. In panel **A**, distributions were relative to the number of tumors within each subgroup; 863 tumors were tBRCAm, 107 were non-BRCA HRRm, and 868 were non-HRRm. The dashed vertical line denotes the GIS cutoff of 42. In panel **B**, tumor HRD status was based on 1788 tumors. HRD-positive was defined as the presence of a tBRCAm and/or a GIS of ≥ 42, HRD-negative as a GIS of < 42 and absence of a tBRCAm, and HRD-unknown were cases where a GIS could not be determined. In panel **C**, the box plot shows median (IQR) and whiskers indicate 1.5 times the IQR above Q3 and below Q1. The dashed horizontal line denotes the GIS cutoff of 42. Tumors with co-occurring *BRCA1* and *BRCA2* mutations were excluded. *BRCA1*m, *BRCA1* mutation; *BRCA2*m, *BRCA2* mutation; BRCAm, *BRCA1* and/or *BRCA2* mutation; GIS, genomic instability score; HRRm, homologous recombination repair mutation; IQR, interquartile range; Q, quartile; tBRCAm, tumor BRCAm

When assessing the distribution of GIS across datasets, three distinct clusters were identified based on BRCA, non-BRCA HRR, and non-HRR mutation status. As detailed above, GIS for tBRCAm tumors was predominantly high, while those patients with non-BRCA HRRm (*n* = 107; median 42 [IQR 29–58]) and those with non-HRRm (*n* = 868; median 32 [IQR 20–55]) had GIS patterns that were variable and predominantly low, respectively (Fig. 3A). Classifying evaluable patients from PAOLA-1, OPINION, LIGHT, and Study 19 (*n* = 1788) according to their HRD and HRR biomarker status demonstrated that HRD and HRRm assays are not interchangeable (Fig. 3B). It should be noted that the SOLO1 and SOLO2 trials were not included in this analysis as these studies only enrolled patients with BRCAm. Therefore, non-BRCA HRRm and non-HRRm status were not evaluable [8, 21]. Of the 1788 tumor samples from PAOLA-1, OPINION, LIGHT, and Study 19 with genomic data, 121 (6.8%) were identified as non-BRCA HRRm, which was smaller than the proportion identified as HRD-positive excluding BRCAm (319; 17.8%) (Fig. 3B). Overall, patients with a non-BRCA HRRm were not enriched in HRD-positive tumors and a similar proportion of non-BRCA HRRm tumors were HRD-positive (GIS ≥ 42 in 55/121; 45.5%) compared with HRD-negative (GIS < 42 in 52/121; 43.0%) or GIS unknown (14/121; 11.6%) states (Fig. 3B).

**Fig. 4:**
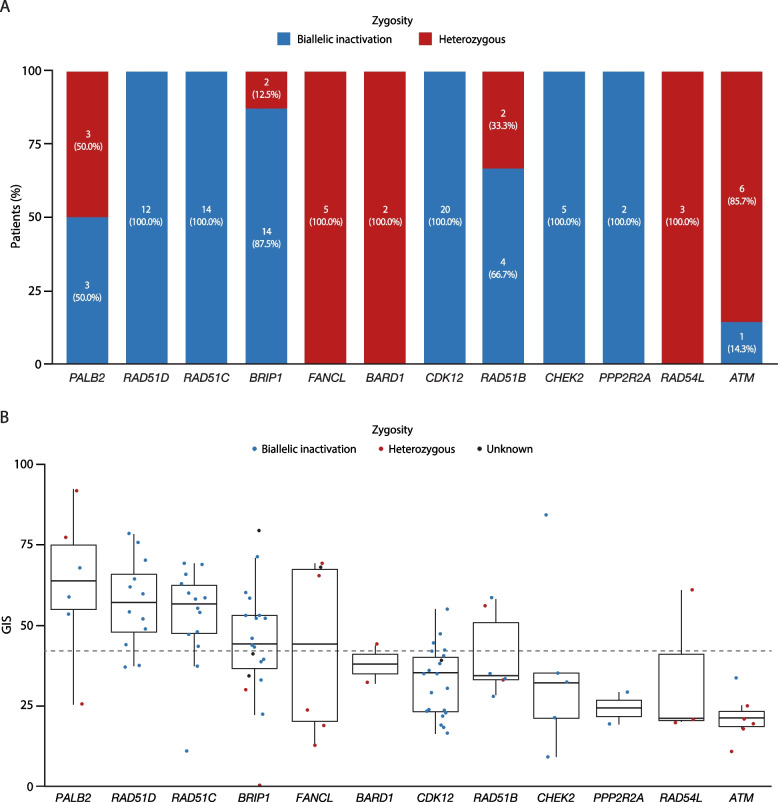
**A** Gene-specific zygosity and **B** GIS distribution in non-BRCA HRRm tumors. Panel **A** shows results for 98 patients with a non-BRCA HRRm and evaluable zygosity. Excludes tumors with co-occurring HRR, as gene-specific zygosity on the patient level cannot be assessed. Cases where gene-specific zygosity could not be assessed are not shown. In panel **B**, the box plot shows median (IQR) and whiskers indicate 1.5 times the IQR above Q3 and below Q1. The dashed horizontal line denotes the GIS cutoff of 42. Excludes tumors with co-occurring HRR, as gene-specific HRD and zygosity on the patient level cannot be assessed, and samples are only included where a GIS was calculated (103 patient samples). An additional gene (*CHEK1)* is not shown in panels **A** or **B**, as no individual mutations were detected. GIS, genomic instability score; HRD, homologous recombination deficiency; HRR, homologous recombination repair; HRRm, HRR mutation; IQR, interquartile range; Q, quartile

Median GIS for non-BRCA HRRm (median GIS 42 [IQR 29–58]) was significantly lower than that for *BRCA1*m or *BRCA2*m (*P* < 0.001 in both instances) and significantly higher than that for non-HRRm (*P* ~ 0.003) (Fig. 3C). The difference in median GIS for non-BRCA HRRm between tumors from patients in response after first-line platinum-based chemotherapy and those with platinum-sensitive relapsed disease was not statistically significant (*P* = 0.12) (Additional file 1: Fig. S6A). Genomic instability for tumors with a non-BRCA HRRm was also assessed by individual study (Additional file 1: Fig. S6B). The median GIS for non-BRCA HRRm patients exceeded the cutoff for HRD-positivity only in OPINION (median GIS 54; IQR 38–68; *n* = 31) and was lower in PAOLA-1 (median GIS 38; IQR 24–54; *n* = 43), LIGHT (median GIS 38; IQR 23–53; *n* = 22), and Study 19 (median GIS 33; IQR 30–54; *n* = 11). To assess this finding further, we performed gene-by-gene–level analysis within the non-BRCA HRRm group. In relation to gene-specific LOH, *CDK12* (20/20; 100.0%), *BRIP1* (14/16; 87.5%), *RAD51C* (14/14; 100.0%), and *RAD51D* (12/12; 100.0%) genes had high levels of biallelic inactivation; the number of evaluable tumors with a non-BRCA HRRm other than in these genes was too low to identify any trends in gene-specific LOH (Fig. 4A). GIS was also assessed for individual non-BRCA HRR genes. Tumors with mutations in five non-BRCA HRR genes (*BRIP1*, *RAD51C*, *RAD51D*, *PALB2*, and *FANCL*) had a median GIS ≥ 42 (Fig. 4B). In particular, those with a mutation in *BRIP1* (median GIS 46; IQR 41–58), *RAD51C* (median GIS 58; IQR 48–66), *RAD51D* (median GIS 62; IQR 54–69), and *PALB2* (median GIS 64; IQR 58–74) had a relatively higher median GIS than the other non-BRCA HRR genes (Fig. 4B) such as *CDK12* (median GIS 33; IQR 23–39) (Fig. 4B). The GIS distribution for individual genes was broadly consistent across studies (Additional file 1: Fig. S7); however, mutation frequencies for individual genes were not always consistent between studies and small patient numbers mean that gene-by-gene–level data for individual studies should be interpreted with caution.

### Genomic instability patterns with non-HRRm (gene mutations other than BRCA or non-BRCA HRRm)

Patterns of genomic instability were assessed in patients without BRCAm or non-BRCA HRRm (i.e., the non-HRRm population, *n* = 1005, of whom 868 had evaluable GIS) relative to the BRCAm population.

**Fig. 5:**
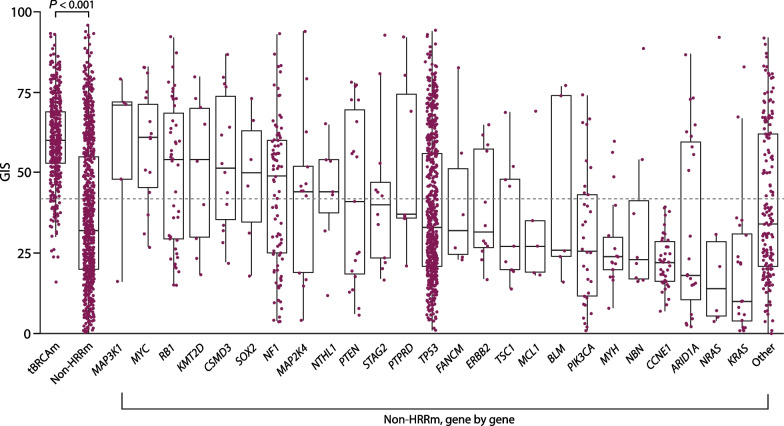
GIS distribution in tumors with tBRCAm and non-HRRm (gene alterations other than tBRCAm or non-BRCA HRRm) in PAOLA-1, OPINION, LIGHT, and Study 19. The box plot shows median (IQR) and whiskers indicate 1.5 times the IQR above Q3 and below Q1. The dashed horizontal line denotes the GIS cutoff of 42. The figure shows results for 1295 patient samples (427 tBRCAm with an evaluable GIS and 868 non-HRRm). Only mutations that qualified as deleterious/suspected deleterious were included in the analysis. ‘Other’ represents all genes that are mutated < 5 times across the datasets. Study 19 data were derived from the FoundationOne® assay results (Foundation Medicine, Inc., Cambridge, MA, USA), rather than Myriad MyChoice® CDx test results. Overall GIS for tBRCAm and non-HRRm are shown for completeness. The figure does not consider co-occurrence with *TP53*. BRCAm, *BRCA1* and/or *BRCA2* mutation; GIS, genomic instability score; HRRm, homologous recombination repair mutation; IQR, interquartile range; Q, quartile; tBRCAm, tumor BRCAm

Overall, non-HRRm tumors had a significantly lower median GIS (median GIS 32 [IQR 20–55]) than BRCAm tumors (*P* < 0.001; Fig. 5). In non-HRRm tumors, some individual genes had a high (≥ 42) median GIS (i.e., *MAP3K1*, *MYC, RB1*, *KMT2D*, *CSMD3*, *SOX2*, *NF1*, *MAP2K4*, *NTHL1*) and some had a low (< 42) median GIS (i.e., *PTEN*, *STAG2*, *PTPRD*, *FANCM*, *ERBB2*, *TSC1*, *MCL1*, *BLM*, *PIK3CA*, *MYH*, *NBN*, *CCNE1*, *ARID1A*, *NRAS*, *KRAS*). On a gene-by-gene level, patterns of GIS were generally stochastic and/or limited in sample size. However, some select genes showed consistent patterns.

**Fig. 6:**
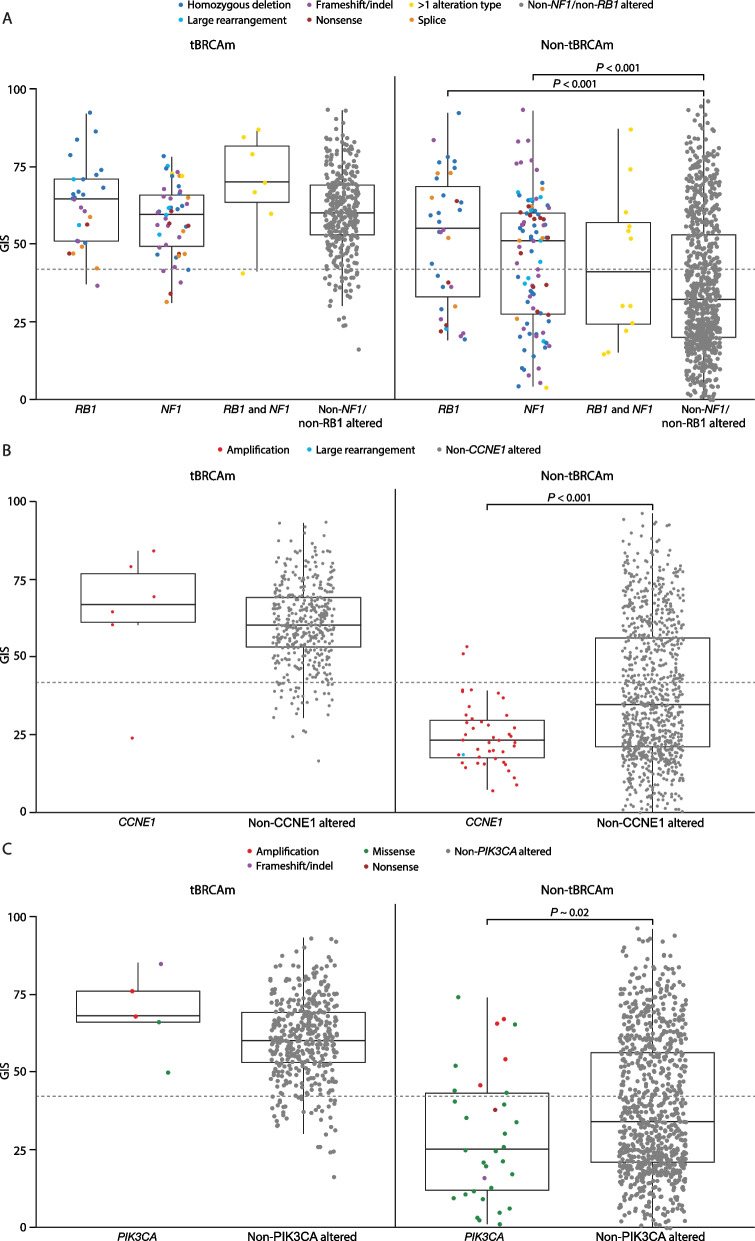
GIS in tumors with **A ***NF1* and *RB1* alterations, **B ***CCNE1* alterations, and **C ***PIK3CA* alterations according to tBRCAm and non-tBRCAm status. The box plot shows median (IQR) and whiskers indicate 1.5 times the IQR above Q3 and below Q1. The dashed horizontal line denotes the GIS cutoff of 42. Figures do not consider co-occurrence with *TP53*. BRCAm, *BRCA1* and/or *BRCA2* mutation; GIS, genomic instability score; IQR, interquartile range; Q, quartile; tBRCA, tumor BRCAm

In non-HRRm tumors, *NF1-* (median GIS 49; IQR 25–60; *n* = 90) and *RB1-* (median GIS 55; IQR 30–71; *n* = 45) altered tumors had a high GIS (Fig. 6A). In all patients assessed, *NF1* and *RB1* mutations co-occurred with BRCAm in 33.5% (55/164) and 44.3% (39/88) of cases, respectively. Although both *NF1* and *RB1* mutations are known to co-occur with BRCAm (where GIS is predominantly high) [24], median GIS was high in tumors with *NF1* and *RB1* mutations in both BRCAm (*RB1*, median GIS 65; IQR 51–71; *NF1*, median GIS 59; IQR 48–66) and non-BRCAm (*RB1*, median GIS 55; IQR 33–69; *NF1*, median GIS 50; IQR 28–60) states, across gene alteration subtypes. GIS was significantly higher for both *RB1*-mutated and *NF1*-mutated tumors relative to non-*NF1-*/non-*RB1*-mutated tumors (*P* < 0.001 in both cases) in non-BRCAm states (Fig. 6A). *RB1* and *NF1* alterations also co-occurred with each other in these cohorts at a rate of 1.4% (*n* = 7) and 1.3% (*n* = 13) in BRCAm and non-BRCAm tumors, respectively.

Conversely, in all patients assessed, tumors with *CCNE1* alterations (*n* = 49) had a low GIS (median 24; IQR 18–29), were predominantly non-BRCAm (43/49; 87.8%), and had a significantly lower GIS than non-*CCNE1*-mutated tumors (*P* < 0.001) in non-BRCAm states (Fig. 6B). A similar pattern was also observed for *PIK3CA*-mutated tumors (*n* = 43) (median 32, IQR 14–52), which were predominantly non-BRCAm (38 of 43; 88.4%), where a significantly lower GIS was seen with *PIK3CA*-mutated versus non-*PIK3CA*-mutated tumors (*P* ~ 0.02) in non-BRCAm states (Fig. 6C).

The pattern of gene alterations reported in PAOLA-1, OPINION, LIGHT, and Study 19 in the context of BRCAm, HRD, and non-BRCA HRRm is shown in Additional file 1: Fig. S8.

## Discussion

We analyzed genomic instability data from six phase II or III studies evaluating olaparib as maintenance therapy (SOLO1 [8, 13], PAOLA-1 [9, 22], Study 19 [14–16], SOLO2 [18, 21], OPINION [19, 20]) or as treatment (LIGHT [12, 17]). Our study comprehensively describes the relationship between GIS and a 108-gene panel in > 2000 patients with newly diagnosed [8, 9, 13, 22] or platinum-sensitive relapsed [12, 14–21] high-grade serous or high-grade endometrioid ovarian cancer, and to our knowledge represents the largest combined HRD analysis of high-grade epithelial ovarian cancer using a commercially available HRD assay. To our knowledge, for the first time, our study shows the relationship between GIS and a broad panel of genes in carefully curated patients without BRCAm or HRRm.

We demonstrated that GIS has an overall bimodal distribution in high-grade serous or high-grade endometrioid ovarian cancer, with high GIS in BRCAm tumors, variable GIS in non-BRCA HRRm tumors (possibly reflecting differences in the role of these genes in the HRR pathway), and predominantly low GIS in non-HRRm tumors. This pattern was consistent across individual studies and in patients with newly diagnosed ovarian cancer or PSROC.

In our analysis and consistent with the individual studies [7, 25, 26], BRCAm was associated with high GIS and high rates of biallelic loss, irrespective of BRCAm being germline or somatic in origin. Our analysis also demonstrated that GIS was high regardless of mutation subtype. Although both *BRCA1*m and *BRCA2*m tumors had a very high GIS (with a similar IQR), a significantly higher score was observed for those with *BRCA1*m. Clinical trial data have demonstrated significant benefit of PARP inhibitor treatment in ovarian cancer patients with BRCAm; however, some studies suggest that patients with *BRCA2*m have relatively greater sensitivity to PARP inhibition than those with *BRCA1*m [27, 28]. In the SOLO1 trial, benefit from olaparib was demonstrated in patients with a BRCAm regardless of whether genome-wide LOH scores were high or low [25]. These apparently discordant observations may reflect the fact that GIS is only a surrogate marker for sensitivity to PARP inhibition and not the sole determinant. It is possible that once the threshold for GIS has been passed, other factors, such as drug resistance mechanisms, play a more prominent role in tumor sensitivity to PARP inhibition. Further work is needed to understand the relationship between BRCAm and GIS in BRCAm tumors with low genomic instability.

As shown in phase III trials [9–11], HRD, as determined by genomic instability testing, is critical in the newly diagnosed ovarian cancer setting to identify which patients may experience the greatest benefit from PARP inhibitor maintenance therapy [29, 30]. HRD is a measure of global genomic instability induced by defects in the HRR pathway, while HRRm reflects the presence of mutations in specific genes involved in the HRR pathway. Our data demonstrate that HRD and HRRm analyses are not interchangeable and identify different sub-populations of patients. The finding that there was no statistically significant difference in median GIS across the non-BRCA HRRm population in the newly diagnosed ovarian cancer and PSROC settings was unexpected as it suggests that a difference in GIS does not explain why HRRm was predictive of PARP inhibitor benefit in Study 19 [7], but not in PAOLA-1 [31]. Some selection for patients with an overall higher GIS might have been expected in the platinum-sensitive relapsed setting given these studies only included patients known to have platinum-sensitive disease, whereas patients with no evidence of disease following cytoreductive surgery and whose sensitivity to platinum was unknown were eligible for inclusion in the first-line studies. Non-BRCA HRRm were low in prevalence, not enriched in HRD-positive tumors, and heterogeneous with regards to biallelic loss and GIS. *RAD51C*, *RAD51D*, *BRIP1*, and *PALB2* are genes known to be associated with a hereditary risk of ovarian cancer [32–34]. The small number of *PALB2* mutations limits interpretation; however, *RAD51C*, *RAD51D*, and *BRIP1* mutations were associated with both high rates of biallelic loss and high GIS, suggesting that these genes might be true drivers of HRD in ovarian cancer. Mutations in these genes accounted for the majority of non-BRCA HRRm in OPINION, compared with PAOLA-1, LIGHT, and Study 19 where other non-BRCA HRRm genes were also prevalent. This could explain why we observed a higher degree of GIS in OPINION relative to the other studies. In terms of other non-BRCA HRR genes, studies suggest that loss-of-function mutations in *CDK12* confer sensitivity to PARP inhibition [35]. A possible explanation for the relatively low GIS associated with *CDK12* in this analysis is that *CDK12* mutations have a distinct genomic instability pattern characterized by focal tandem repeats [36] and the Myriad MyChoice® CDx assay does not detect this particular genomic instability signature. Our knowledge of what constitutes an HRRm gene continues to evolve. Only two patients with variants in *PPP2R2A* were included in this analysis; low GIS was observed in both cases. After the studies included in this analysis were initiated, data from the PROfound study demonstrated that no benefit of olaparib over control therapy was observed in patients with prostate cancer and *PPP2R2A* mutations and was not predictive of benefit from PARP inhibitor therapy [37].

In terms of alterations outside of BRCAm or HRRm, those in *MAP3K1*, *MYC*, *RB1*, *KMT2D*, *CSMD3*, *SOX2*, *NF1*, *MAP2K4*, and *NTHL1* had a high median GIS and those in *PTEN*, *STAG2*, *PTPRD*, *FANCM*, *ERBB2*, *TSC1*, *MCL1*, *BLM*, *PIK3CA*, *MYH*, *NBN*, *CCNE1*, *ARID1A*, *NRAS*, and *KRAS* had a low median GIS. It is now understood that *RB1* loss is not mutually exclusive with HRRm, and improved outcomes have been reported when *RB1* loss co-occurs with HRRm [38, 39]. This analysis demonstrates that even in tumors without BRCAm or HRRm, *NF1* and *RB1* mutations were associated with relatively high genomic instability; further work is needed to understand the mechanistic relationship between *NF1* and *RB1* and GIS. By contrast, mutations in *CCNE1* and *PIK3CA* were associated with relatively low genomic instability that was found to be significantly lower than that seen in non-*CCNE1*-altered and non-*PIK3CA*-mutated tumors, respectively, in non-BRCAm states. To our knowledge, *PIK3CA* mutations have not previously been shown to be associated with low GIS and an association between *CCNE1* amplification and low GIS has previously only been observed in smaller datasets [40]. Increased cyclin E expression, either by *CCNE1* gene amplification, copy-number gain, or elevated protein expression, is associated with poor clinical outcomes and resistance to DNA-damaging drugs in ovarian cancer [24, 41]. For example, *CCNE1* amplification was correlated with shorter relapse-free survival in patients with ovarian carcinomas treated with platinum-based chemotherapy [41]. The results of our analysis and others [42, 43] suggest that patients with *CCNE1* amplifications (but without BRCAm) may potentially benefit from alternative targeted therapies. *PIK3CA* mutations are also associated with poor response to platinum-based chemotherapy and platinum resistance in patients with ovarian cancer [44]. Patients with alterations in *CCNE1* or *PIK3CA* therefore represent a population with very high unmet medical needs. The optimal treatment of *CCNE1*-amplified and *PIK3CA*-mutated tumors warrants further investigation; the PI3K/AKT/mTOR pathway is one of the potential therapeutic targets for *CCNE1*-mutated [45] and *PIK3CA*-mutated [44] tumors.

Ovarian cancer is the archetypal homologous recombination deficient tumor and is the only tumor type to date where genomic instability status is predictive of benefit from PARP inhibitor therapy. Although correlating genomic instability with clinical benefit was beyond the scope of the current analysis, previous analyses of PAOLA-1 [9, 22], Study 19 [7], OPINION [19, 20], and LIGHT [12, 17] have evaluated clinical outcome according to HRD status. Further work is needed to understand the clinical implications of these data and to evaluate potential links between genomic instability and clinical benefit from PARP inhibitor therapy for individual non-BRCA HRR and non-HRR genes. To date, post hoc exploratory analysis found that non-BRCA HRR gene panels were not predictive of the efficacy of maintenance olaparib plus bevacizumab in PAOLA-1 [31]. By contrast, subgroup analyses based on non-BRCA HRRm demonstrated the benefit of olaparib treatment in Study 19 [7], olaparib activity comparable with that seen in BRCAm in the ORZORA trial [46], and longer PFS benefit in patients with non-BRCA HRRm relative to patients with non-HRRm in the OPINION trial [47]. One explanation for these apparent discrepancies might be a difference in signal between the first-line [31] and relapsed disease [7] settings, with the relapsed disease population more likely to be enriched for platinum sensitivity. These studies included small numbers of individual non-BRCA HRR genes and differences between the studies in gene-to-gene prevalence may be another potential explanation for apparent discrepancies. For example, of the non-BRCA HRRm subgroup, *CDK12*, *BRIP1*, and *RAD51C* accounted for 24%, 13%, and 17%, respectively, in PAOLA-1 [31]; 36%, 15%, and 18%, respectively, in ORZORA [46]; and 9%, 21%, and 24%, respectively, in OPINION [47]. Furthermore, these studies were not individually powered to assess the predictive power of these non-BRCA HRR genes, making comparisons difficult. In terms of other PARP inhibitors, *RAD51C* and *RAD51D* mutations predicted response to treatment with rucaparib patients with relapsed ovarian cancer in a post hoc exploratory analysis of ARIEL2 [48]. Although we found *BRIP1* mutations had a high GIS, they are yet to be established as predictive of PARP inhibitor response [31]. Better understanding of drivers of genomic instability in ovarian cancer may open up opportunities for PARP inhibitor use in other indications where GIS alone has not been shown to be predictive. Our study identified a further nine genes (*MAP3K1*, *MYC*, *RB1*, *KMT2D*, *CSMD3*, *SOX2*, *NF1*, *MAP2K4*, and *NTHL1*) which, when mutated, were associated with high median GIS and an additional 15 genes (*PTEN*, *STAG2*, *PTPRD*, *FANCM*, *ERBB2*, *TSC1*, *MCL1*, *BLM*, *PIK3CA*, *MYH*, *NBN*, *CCNE1*, *ARID1A*, *NRAS*, and *KRAS*) which, when mutated, were associated with low median GIS; some of these genes (e.g., *CCNE1*, *ARID1A*, *NRAS*, and *KRAS*) are linked with replication stress. Thus, our data highlight the heterogeneity of HRD-positive and HRD-negative populations, which appear to have different biology, and the need to better personalize treatment options that target alternative pathways. Further clinical studies would be required to validate these targets as biomarkers for PARP inhibitor benefit in ovarian cancer, which is beyond the scope of this study. These data also signal the need to explore PARP inhibitor benefit in other tumor types harboring mutations shown here to associate with high GIS and, potentially, an HRD phenotype.

This analysis is associated with several limitations. The first is pooling data from six different trials—with variations in study design, treatment setting, and patient selection criteria as well as diagnostic tests applied—which may have contributed to differences in the results between studies. For example, these data do not represent the patterns of genomic instability in an all-comer population, as some of the trials selected for biomarkers prospectively (OPINION [19, 20], SOLO1 [8, 13], SOLO2 [18, 21], LIGHT [12, 17]), whereas others did not (PAOLA-1 [9, 22], Study 19 [14–16]). Hence, the prevalence of BRCAm, HRRm, and non-HRRm is not reflective of all patients with ovarian cancer. In addition, LIGHT was conducted in the later-line treatment setting in patients who had received one or more prior lines of platinum-based chemotherapy [12, 17], whereas the other studies evaluated olaparib maintenance therapy in patients who responded to platinum-based chemotherapy (in combination with bevacizumab in PAOLA-1 [9, 22]). It is not clear to what extent this heterogeneity may have impacted the pooled dataset used in this analysis. Secondly, while *BRCA1* methylation data are available from Study 19 [7] and PAOLA-1 [49], methylation data were not available from the other studies included in this analysis. Hypermethylation may partly explain the genomic instability seen in some non-HRRm tumors. *BRCA1* hypermethylation is found in approximately 10% of ovarian tumors [50] and is associated with high levels of genomic instability [7]. In PAOLA-1, of the 72 *BRCA1* or *RAD51C* methylated tumors with a valid GIS, 92% had a GIS ≥ 42 [49]. Similarly, a recent study in high-grade serous ovarian carcinoma showed that homozygous methylation of the *RAD51C* promoter is predictive of sensitivity to PARP inhibition [51]. Lack of methylation data was a limitation when assessing genomic instability in the non-BRCAm non-HRRm subgroup. A further limitation is the possibility that some large rearrangements in BRCA (germline or somatic) and some *CCNE1* amplifications may not have been detected because of the assays used, causing tumors with these alterations to be underrepresented in the analysis. In addition, data regarding whole-genome duplication were not available for this analysis; however, minor allele frequency was assessed to ensure it was at zero when evaluating biallelic loss. Finally, this analysis only includes clinical trials of olaparib and may not be representative of all PARP inhibitors.

In summary, this analysis of genomic data from six olaparib studies [8, 9, 12–22], including > 2000 patients with ovarian cancer, reveals distinct patterns of genomic instability in this patient population. We demonstrate that GIS has an overall bimodal distribution in ovarian cancer, with germline and somatic BRCAm tumors typically having a high GIS and high biallelic loss. We show that non-BRCA HRRm are low in prevalence and heterogeneous with regards to biallelic loss and GIS. Non-BRCA HRR genes that were associated with high GIS included *BRIP1*, *RAD51C*, and *RAD51D*. In terms of mutations outside of BRCAm or HRRm, *NF1* and *RB1* mutations had a relatively high GIS, and *CCNE1*-amplified and *PIK3CA*-mutated tumors had a low GIS and were predominantly non-BRCAm.

## Conclusion

This analysis of data from six studies evaluating olaparib as maintenance therapy or treatment provides valuable insight into patterns of genomic instability and potential drivers of HRD, other than BRCAm, among patients with ovarian cancer. These data will help guide future research into the potential clinical effectiveness of anti-cancer treatments in ovarian cancer, including PARP inhibitors as well as other precision oncology agents.

## Supplementary Information

Additional file 1: Table S1. Summary of ovarian cancer trials included in this analysis. Table S2. Assessment of mutation status and GIS in the ovarian cancer trials used in this analysis. Table S3. Full gene panel included in the Myriad tumor tissue test^a^ (Myriad Genetic Laboratories, Inc.). Figure S1. GIS distribution by patient race in (A) all patients and (B) patients with a tBRCAm, by tumor histology in (C) all patients and (D) patients with a tBRCAm, and by primary tumor location in (E) all patients and (F) patients with a tBRCAm. Figure S2. GIS distribution in tumors with and without tBRCAm by individual study in PAOLA-1, OPINION, LIGHT, Study 19, SOLO1, and SOLO2. Figure S3. GIS distribution in tumors from patients (A) in response after first-line platinum-based chemotherapy and (B) with platinum-sensitive relapsed disease, and (C) GIS distribution by germline and somatic tumor BRCAm status and in non-tBRCAm tumors. Figure S4. (A) Gene-specific zygosity in tumors with* BRCA1*m or *BRCA2*m, (B) gene-specific zygosity and the rate of biallelic loss in tumors with a BRCAm, and (C) gene-specific zygosity and the rate of biallelic loss in tumors with germline *BRCA1*m or *BRCA2*m and somatic *BRCA1*m or *BRCA2*m. Figure S5. Gene-specific zygosity in patients with *BRCA1*m or *BRCA2*m by individual study. Figure S6. GIS distribution in (A) non-BRCA HRRm tumors from patients in response after first-line platinum-based chemotherapy and with platinum-sensitive relapsed disease and (B) in tumors with non-BRCA HRRm by individual study in PAOLA-1, OPINION, LIGHT, and Study 19. Figure S7. GIS distribution in patients with non-BRCA HRRm by individual study (PAOLA-1, OPINION, LIGHT, and Study 19). Figure S8. Genomic alterations detected in PAOLA-1, OPINION, LIGHT, and Study 19.

## Data Availability

The data generated in this study are not publicly available because of patient privacy but are available upon reasonable request in accordance with AstraZeneca’s data sharing policy described at https://astrazenecagrouptrials.pharmacm.com/ST/Submission/Disclosure. Data for studies directly listed on Vivli can be requested through Vivli at www.vivli.org. The request will undergo an internal review process, and, if approved, data will be prepared and shared with specified accessors named on the request form for 12 months via Vivli Secure Research Environment. Data for studies not listed on Vivli could be requested through Vivli at https://vivli.org/members/enquiries-about-studies-not-listed-on-the-vivli-platform/. AstraZeneca Vivli member page is also available outlining further details: https://vivli.org/ourmember/astrazeneca/. The code generated in this work is available on GitHub (https://github.com/AstraZeneca/Ovarian_genomics_HRD) [52].

## Abbreviations

BRCA: *BRCA1* and/or *BRCA2*
BRCAm: *BRCA1* and/or *BRCA2* mutation
CI: Confidence interval
GIS: Genomic instability score
HR: Hazard ratio
HRD: Homologous recombination deficiency
HRR: Homologous recombination repair
HRRm: Homologous recombination repair mutation
IQR: Interquartile range
LST: Large-scale state transition
LOH: Loss of heterozygosity
PARP: Poly(ADP-ribose) polymerase
PSROC: Platinum-sensitive relapsed ovarian cancer
TAI: Telomeric allelic imbalance
tBRCAm: Tumor BRCAm

## References

[1] Stewart MD, Merino Vega D, Arend RC, Baden JF, Barbash O, Beaubier N, et al. Homologous recombination deficiency: concepts, definitions, and assays. Oncologist. 2022;27(3):167–74. 35274707 10.1093/oncolo/oyab053 PMC8914493

[2] Abkevich V, Timms KM, Hennessy BT, Potter J, Carey MS, Meyer LA, et al. Patterns of genomic loss of heterozygosity predict homologous recombination repair defects in epithelial ovarian cancer. Br J Cancer. 2012;107(10):1776–82. 23047548 10.1038/bjc.2012.451 PMC3493866

[3] Birkbak NJ, Wang ZC, Kim JY, Eklund AC, Li Q, Tian R, et al. Telomeric allelic imbalance indicates defective DNA repair and sensitivity to DNA-damaging agents. Cancer Discov. 2012;2(4):366–75. 22576213 10.1158/2159-8290.CD-11-0206 PMC3806629

[4] Popova T, Manié E, Rieunier G, Caux-Moncoutier V, Tirapo C, Dubois T, et al. Ploidy and large-scale genomic instability consistently identify basal-like breast carcinomas with BRCA1/2 inactivation. Cancer Res. 2012;72(21):5454–62. 22933060 10.1158/0008-5472.CAN-12-1470

[5] O’Connor MJ. Targeting the DNA damage response in cancer. Mol Cell. 2015;60(4):547–60. 26590714 10.1016/j.molcel.2015.10.040

[6] Rudolph J, Jung K, Luger K. Inhibitors of PARP: number crunching and structure gazing. Proc Natl Acad Sci U S A. 2022;119(11): e2121979119. 35259019 10.1073/pnas.2121979119 PMC8931346

[7] Hodgson DR, Dougherty BA, Lai Z, Fielding A, Grinsted L, Spencer S, et al. Candidate biomarkers of PARP inhibitor sensitivity in ovarian cancer beyond the BRCA genes. Br J Cancer. 2018;119(11):1401–9. 30353044 10.1038/s41416-018-0274-8 PMC6265286

[8] Moore K, Colombo N, Scambia G, Kim B-G, Oaknin A, Friedlander M, et al. Maintenance olaparib in patients with newly diagnosed advanced ovarian cancer. N Engl J Med. 2018;379(26):2495–505. 30345884 10.1056/NEJMoa1810858

[9] Ray-Coquard I, Pautier P, Pignata S, Pérol D, González-Martín A, Berger R, et al. Olaparib plus bevacizumab as first-line maintenance in ovarian cancer. N Engl J Med. 2019;381(25):2416–28. 31851799 10.1056/NEJMoa1911361

[10] González-Martín A, Pothuri B, Vergote I, DePont CR, Graybill W, Mirza MR, et al. Niraparib in patients with newly diagnosed advanced ovarian cancer. N Engl J Med. 2019;381(25):2391–402. 31562799 10.1056/NEJMoa1910962

[11] Monk BJ, Parkinson C, Lim MC, O’Malley DM, Oaknin A, Wilson MK, et al. A randomized, phase III trial to evaluate rucaparib monotherapy as maintenance treatment in patients with newly diagnosed ovarian cancer (ATHENA-MONO/GOG-3020/ENGOT-ov45). J Clin Oncol. 2022;40(34):3952–64. 35658487 10.1200/JCO.22.01003 PMC9746782

[12] Cadoo K, Simpkins F, Mathews C, Liu YL, Provencher D, McCormick C, et al. Olaparib treatment for platinum-sensitive relapsed ovarian cancer by BRCA mutation and homologous recombination deficiency status: phase II LIGHT study primary analysis. Gynecol Oncol. 2022;166(3):425–31. 35803835 10.1016/j.ygyno.2022.06.017 PMC9909678

[13] DiSilvestro P, Banerjee S, Colombo N, Scambia G, Kim BG, Oaknin A, et al. Overall survival with maintenance olaparib at a 7-year follow-up in patients with newly diagnosed advanced ovarian cancer and a BRCA mutation: the SOLO1/GOG 3004 trial. J Clin Oncol. 2023;41(3):609–17. 36082969 10.1200/JCO.22.01549 PMC9870219

[14] Friedlander M, Matulonis U, Gourley C, du Bois A, Vergote I, Rustin G, et al. Long-term efficacy, tolerability and overall survival in patients with platinum-sensitive, recurrent high-grade serous ovarian cancer treated with maintenance olaparib capsules following response to chemotherapy. Br J Cancer. 2018;119(9):1075–85. 30353045 10.1038/s41416-018-0271-y PMC6219499

[15] Ledermann J, Harter P, Gourley C, Friedlander M, Vergote I, Rustin G, et al. Olaparib maintenance therapy in platinum-sensitive relapsed ovarian cancer. N Engl J Med. 2012;366(15):1382–92. 22452356 10.1056/NEJMoa1105535

[16] Ledermann J, Harter P, Gourley C, Friedlander M, Vergote I, Rustin G, et al. Olaparib maintenance therapy in patients with platinum-sensitive relapsed serous ovarian cancer: a preplanned retrospective analysis of outcomes by BRCA status in a randomised phase 2 trial. Lancet Oncol. 2014;15(8):852–61. 24882434 10.1016/S1470-2045(14)70228-1

[17] Mathews CA, Simpkins F, Cadoo KA, Liu YL, Provencher DM, McCormick C, et al. Olaparib treatment (Tx) in patients (pts) with platinum-sensitive relapsed ovarian cancer (PSR OC) by BRCA mutation (BRCAm) and homologous recombination deficiency (HRD) status: overall survival (OS) results from the phase II LIGHT study. J Clin Oncol. 2021;39(15_suppl):5515.

[18] Poveda A, Floquet A, Ledermann JA, Asher R, Penson RT, Oza AM, et al. Olaparib tablets as maintenance therapy in patients with platinum-sensitive relapsed ovarian cancer and a BRCA1/2 mutation (SOLO2/ENGOT-Ov21): a final analysis of a double-blind, randomised, placebo-controlled, phase 3 trial. Lancet Oncol. 2021;22(5):620–31. 33743851 10.1016/S1470-2045(21)00073-5

[19] Poveda A, Lheureux S, Colombo N, Cibula D, Lindemann K, Weberpals J, et al. Olaparib maintenance monotherapy in platinum-sensitive relapsed ovarian cancer patients without a germline BRCA1/BRCA2 mutation: OPINION primary analysis. Gynecol Oncol. 2022;164(3):498–504. 35063276 10.1016/j.ygyno.2021.12.025

[20] Poveda A, Lheureux S, Colombo N, Cibula D, Elstrand M, Weberpals J, et al. 531P maintenance olaparib monotherapy in patients (pts) with platinum-sensitive relapsed ovarian cancer (PSR OC) without a germline BRCA1/BRCA2 mutation (non-gBRCAm): final overall survival (OS) results from the OPINION trial. Ann Oncol. 2022;33:S790.

[21] Pujade-Lauraine E, Ledermann JA, Selle F, Gebski V, Penson RT, Oza AM, et al. Olaparib tablets as maintenance therapy in patients with platinum-sensitive, relapsed ovarian cancer and a BRCA1/2 mutation (SOLO2/ENGOT-Ov21): a double-blind, randomised, placebo-controlled, phase 3 trial. Lancet Oncol. 2017;18(9):1274–84. 28754483 10.1016/S1470-2045(17)30469-2

[22] Ray-Coquard I, Leary A, Pignata S, Cropet C, Gonzalez-Martin A, Marth C, et al. Olaparib plus bevacizumab first-line maintenance in ovarian cancer: final overall survival results from the PAOLA-1/ENGOT-ov25 trial. Ann Oncol. 2023;34(8):681–92. 37211045 10.1016/j.annonc.2023.05.005

[23] Telli ML, Timms KM, Reid J, Hennessy B, Mills GB, Jensen KC, et al. Homologous recombination deficiency (HRD) score predicts response to platinum-containing neoadjuvant chemotherapy in patients with triple-negative breast cancer. Clin Cancer Res. 2016;22(15):3764–73. 26957554 10.1158/1078-0432.CCR-15-2477 PMC6773427

[24] Patch AM, Christie EL, Etemadmoghadam D, Garsed DW, George J, Fereday S, et al. Whole-genome characterization of chemoresistant ovarian cancer. Nature. 2015;521(7553):489–94. 26017449 10.1038/nature14410

[25] Gourley C, Brown JS, Lai Z, Lao-Sirieix P, Elks CE, McGarvey H, et al. Analysis of tumour samples from SOLO1: frequency of BRCA specific loss of heterozygosity (LOH) and progression-free survival (PFS) according to homologous recombination repair deficiency (HRD)-LOH score. Ann Oncol. 2019;30(Suppl 5):abstract 998PD.

[26] Hodgson DR, Brown JS, Dearden SP, Lai Z, Elks CE, Milenkova T, et al. Concordance of BRCA mutation detection in tumor versus blood, and frequency of bi-allelic loss of BRCA in tumors from patients in the phase III SOLO2 trial. Gynecol Oncol. 2021;163(3):563–8. 34742578 10.1016/j.ygyno.2021.10.002

[27] Banerjee S, Moore KN, Colombo N, Scambia G, Kim BG, Oaknin A, et al. Maintenance olaparib for patients with newly diagnosed advanced ovarian cancer and a BRCA mutation (SOLO1/GOG 3004): 5-year follow-up of a randomised, double-blind, placebo-controlled, phase 3 trial. Lancet Oncol. 2021;22(12):1721–31. 34715071 10.1016/S1470-2045(21)00531-3

[28] Lorusso D, Lotz JP, Harter P, Cropet C, Pérez MJR, Schauer C, et al. Maintenance olaparib plus bevacizumab (bev) after platinum-based chemotherapy plus bev in patients (pts) with newly diagnosed advanced high-grade ovarian cancer (HGOC): efficacy by BRCA1 or BRCA2 mutation in the phase III PAOLA-1 trial. J Clin Oncol. 2020;38(15_suppl):6039.

[29] Miller RE, Leary A, Scott CL, Serra V, Lord CJ, Bowtell D, et al. ESMO recommendations on predictive biomarker testing for homologous recombination deficiency and PARP inhibitor benefit in ovarian cancer. Ann Oncol. 2020;31(12):1606–22. 33004253 10.1016/j.annonc.2020.08.2102

[30] Vergote I, González-Martín A, Ray-Coquard I, Harter P, Colombo N, Pujol P, et al. European experts consensus: BRCA/homologous recombination deficiency testing in first-line ovarian cancer. Ann Oncol. 2022;33(3):276–87. 34861371 10.1016/j.annonc.2021.11.013

[31] Pujade-Lauraine E, Brown J, Barnicle A, Wessen J, Lao-Sirieix P, Criscione SW, et al. Homologous recombination repair gene mutations to predict olaparib plus bevacizumab efficacy in the first-line ovarian cancer PAOLA-1/ENGOT-ov25 trial. JCO Precis Oncol. 2023;7: e2200258. 36716415 10.1200/PO.22.00258 PMC9928987

[32] Song H, Dicks E, Ramus SJ, Tyrer JP, Intermaggio MP, Hayward J, et al. Contribution of germline mutations in the RAD51B, RAD51C, and RAD51D genes to ovarian cancer in the population. J Clin Oncol. 2015;33(26):2901–7. 26261251 10.1200/JCO.2015.61.2408 PMC4554751

[33] Weber-Lassalle N, Hauke J, Ramser J, Richters L, Groß E, Blümcke B, et al. BRIP1 loss-of-function mutations confer high risk for familial ovarian cancer, but not familial breast cancer. Breast Cancer Res. 2018;20(1):7. 29368626 10.1186/s13058-018-0935-9 PMC5784717

[34] Yang X, Leslie G, Doroszuk A, Schneider S, Allen J, Decker B, et al. Cancer risks associated with germline PALB2 pathogenic variants: an international study of 524 families. J Clin Oncol. 2020;38(7):674–85. 31841383 10.1200/JCO.19.01907 PMC7049229

[35] Bajrami I, Frankum JR, Konde A, Miller RE, Rehman FL, Brough R, et al. Genome-wide profiling of genetic synthetic lethality identifies CDK12 as a novel determinant of PARP1/2 inhibitor sensitivity. Cancer Res. 2014;74(1):287–97. 24240700 10.1158/0008-5472.CAN-13-2541 PMC4886090

[36] Popova T, Manie E, Boeva V, Battistella A, Goundiam O, Smith NK, et al. Ovarian cancers harboring inactivating mutations in CDK12 display a distinct genomic instability pattern characterized by large tandem duplications. Cancer Res. 2016;76(7):1882–91. 26787835 10.1158/0008-5472.CAN-15-2128

[37] Hussain M, Mateo J, Fizazi K, Saad F, Shore N, Sandhu S, et al. Survival with olaparib in metastatic castration-resistant prostate cancer. N Engl J Med. 2020;383(24):2345–57. 32955174 10.1056/NEJMoa2022485

[38] Garsed DW, Alsop K, Fereday S, Emmanuel C, Kennedy CJ, Etemadmoghadam D, et al. Homologous recombination DNA repair pathway disruption and retinoblastoma protein loss are associated with exceptional survival in high-grade serous ovarian cancer. Clin Cancer Res. 2018;24(3):569–80. 29061645 10.1158/1078-0432.CCR-17-1621

[39] Hollis RL, Meynert AM, Michie CO, Rye T, Churchman M, Hallas-Potts A, et al. Multiomic characterization of high-grade serous ovarian carcinoma enables high-resolution patient stratification. Clin Cancer Res. 2022;28(16):3546–56. 35696721 10.1158/1078-0432.CCR-22-0368 PMC9662902

[40] Stronach EA, Paul J, Timms KM, Hughes E, Brown K, Neff C, et al. Biomarker assessment of HR deficiency, tumor BRCA1/2 mutations, and CCNE1 copy number in ovarian cancer: associations with clinical outcome following platinum monotherapy. Mol Cancer Res. 2018;16(7):1103–11. 29724815 10.1158/1541-7786.MCR-18-0034

[41] Nakayama N, Nakayama K, Shamima Y, Ishikawa M, Katagiri A, Iida K, et al. Gene amplification CCNE1 is related to poor survival and potential therapeutic target in ovarian cancer. Cancer. 2010;116(11):2621–34. 20336784 10.1002/cncr.24987

[42] Konstantinopoulos PA, Lheureux S, Moore KN. PARP inhibitors for ovarian cancer: current indications, future combinations, and novel assets in development to target DNA damage repair. Am Soc Clin Oncol Educ Book. 2020;40:1–16. 32364757 10.1200/EDBK_288015

[43] Wilson AJ, Sarfo-Kantanka K, Barrack T, Steck A, Saskowski J, Crispens MA, et al. Panobinostat sensitizes cyclin E high, homologous recombination-proficient ovarian cancer to olaparib. Gynecol Oncol. 2016;143(1):143–51. 27444036 10.1016/j.ygyno.2016.07.088 PMC5031537

[44] Kolasa IK, Rembiszewska A, Felisiak A, Ziolkowska-Seta I, Murawska M, Moes J, et al. PIK3CA amplification associates with resistance to chemotherapy in ovarian cancer patients. Cancer Biol Ther. 2009;8(1):21–6. 19029838 10.4161/cbt.8.1.7209

[45] Bai S, Taylor SE, Jamalruddin MA, McGonigal S, Grimley E, Yang D, et al. Targeting therapeutic resistance and multinucleate giant cells in CCNE1-amplified HR-proficient ovarian cancer. Mol Cancer Ther. 2022;21(9):1473–84. 35732503 10.1158/1535-7163.MCT-21-0873 PMC9452459

[46] Pignata S, Oza A, Hall G, Pardo B, Madry R, Cibula D, et al. Maintenance olaparib in patients with platinum-sensitive relapsed ovarian cancer: outcomes by somatic and germline BRCA and other homologous recombination repair gene mutation status in the ORZORA trial. Gynecol Oncol. 2023;172:121–9. 37030280 10.1016/j.ygyno.2023.03.019

[47] Lindemann K, Skof E, Colombo N, Gonzalez-Martin A, Davidson R, Blakeley C, et al. Olaparib maintenance monotherapy for non-germline BRCA1/2-mutated (non-gBRCAm) platinum-sensitive relapsed ovarian cancer (PSR OC): exploratory biomarker analyses of the phase IIIb OPINION study. Ann Oncol. 2021;32(Suppl 5):S738-9; abstract 40P.

[48] Swisher EM, Kwan TT, Oza AM, Tinker AV, Ray-Coquard I, Oaknin A, et al. Molecular and clinical determinants of response and resistance to rucaparib for recurrent ovarian cancer treatment in ARIEL2 (Parts 1 and 2). Nat Commun. 2021;12(1):2487. 33941784 10.1038/s41467-021-22582-6 PMC8093258

[49] Blons H, Abdelli J, Taly V, Mulot C, Puig PL, You B, et al. BRCA1 and RAD51 methylation impact on outcome in patients with advanced ovarian cancer: a PAOLA-1 ancillary study. J Clin Oncol. 2023;41(Suppl 16):abstract 5559.

[50] Cunningham JM, Cicek MS, Larson NB, Davila J, Wang C, Larson MC, et al. Clinical characteristics of ovarian cancer classified by BRCA1, BRCA2, and RAD51C status. Sci Rep. 2014;4:4026. 24504028 10.1038/srep04026 PMC4168524

[51] Nesic K, Kondrashova O, Hurley RM, McGehee CD, Vandenberg CJ, Ho GY, et al. Acquired RAD51C promoter methylation loss causes PARP inhibitor resistance in high-grade serous ovarian carcinoma. Cancer Res. 2021;81(18):4709–22. 34321239 10.1158/0008-5472.CAN-21-0774 PMC12593227

[52] Barnicle A, Ray-Coquard I, Rouleau E, Cadoo K, Simpkins F, Aghajanian C, et al. Ovarian_genomics_HRD. GitHub; 2024. Available from: https://github.com/AstraZeneca/Ovarian_genomics_HRD.

