# Supplementary material for: Patterns of genomic instability in > 2000 patients with ovarian cancer across six clinical trials evaluating olaparib
Source: Genome Med. 2024 Dec 18;16:145. doi: 10.1186/s13073-024-01413-5 (PMC11657106; doi:10.1186/s13073-024-01413-5)
Supplement: Supplementary file 1 — Additional file 1: Table S1. Summary of ovarian cancer trials included in this analysis. Table S2. Assessment of mutation status and GIS in the ovarian cancer trials used in this analysis. Table S3. Full gene panel included in the Myriad tumor tissue testa (Myriad Genetic Laboratories, Inc.). Figure S1. GIS distribution by patient race in (A) all patients and (B) patients with a tBRCAm, by tumor histology in (C) all patients and (D) patients with a tBRCAm, and by primary tumor location in (E) all patients and (F) patients with a tBRCAm. Figure S2. GIS distribution in tumors with and without tBRCAm by individual study in PAOLA-1, OPINION, LIGHT, Study 19, SOLO1, and SOLO2. Figure S3. GIS distribution in tumors from patients (A) in response after first-line platinum-based chemotherapy and (B) with platinum-sensitive relapsed disease, and (C) GIS distribution by germline and somatic tumor BRCAm status and in non-tBRCAm tumors. Figure S4. (A) Gene-specific zygosity in tumors with BRCA1m or BRCA2m, (B) gene-specific zygosity and the rate of biallelic loss in tumors with a BRCAm, and (C) gene-specific zygosity and the rate of biallelic loss in tumors with germline BRCA1m or BRCA2m and somatic BRCA1m or BRCA2m. Figure S5. Gene-specific zygosity in patients with BRCA1m or BRCA2m by individual study. Figure S6. GIS distribution in (A) non-BRCA HRRm tumors from patients in response after first-line platinum-based chemotherapy and with platinum-sensitive relapsed disease and (B) in tumors with non-BRCA HRRm by individual study in PAOLA-1, OPINION, LIGHT, and Study 19. Figure S7. GIS distribution in patients with non-BRCA HRRm by individual study (PAOLA-1, OPINION, LIGHT, and Study 19). Figure S8. Genomic alterations detected in PAOLA-1, OPINION, LIGHT, and Study 19. [file 13073_2024_1413_MOESM1_ESM.pdf]

Patterns of genomic instability in > 2000 patients with ovarian cancer across six clinical trials evaluating olaparib

Barnicle A, Ray-Coquard I, Rouleau E, et al.

This supplementary appendix has been provided by the authors to give readers additional information about their work.

Table of Contents

Introduction 3

PARP inhibitor approval status 3

Supplementary Table S1. Summary of ovarian cancer trials included in this analysis . 4

Methods 7

Supplementary Table S2. Assessment of mutation status and GIS in the ovarian cancer trials used in this analysis 7

Supplementary Table S3. Full gene panel included in the Myriad tumor tissue test[a] (Myriad Genetic Laboratories, Inc.) 9

Results 10

Supplementary Figure S1. GIS distribution by patient race in (A) all patients and (B) patients with a tBRCAm, by tumor histology in (C) all patients and (D) patients with a tBRCAm, and by primary tumor location in (E) all patients and (F) patients with a tBRCAm 10

Supplementary Figure S2. GIS distribution in tumors with and without tBRCAm by individual study in PAOLA-1, OPINION, LIGHT, Study 19, SOLO1, and SOLO2....... 12

Supplementary Figure S3. GIS distribution in tumors from patients (A) in response after first-line platinum-based chemotherapy and (B) with platinum-sensitive relapsed disease, and (C) GIS distribution by germline and somatic tumor BRCAm status and in non-tBRCAm tumors 13

Supplementary Figure S4. (A) Gene-specific zygosity in tumors with *BRCA1*m or *BRCA2*m, (B) gene-specific zygosity and the rate of biallelic loss in tumors with a BRCAm, and (C) gene-specific zygosity and the rate of biallelic loss in tumors with germline *BRCA1*m or *BRCA2*m and somatic *BRCA1*m or *BRCA2*m 16

Supplementary Figure S5. Gene-specific zygosity in patients with *BRCA1*m or *BRCA2*m by individual study 18

Supplementary Figure S6. GIS distribution in (A) non-BRCA HRRm tumors from patients in response after first-line platinum-based chemotherapy and with platinum-sensitive relapsed disease and (B) in tumors with non-BRCA HRRm by individual study in PAOLA-1, OPINION, LIGHT, and Study 19 19

Supplementary Figure S7. GIS distribution in patients with non-BRCA HRRm by individual study (PAOLA-1, OPINION, LIGHT, and Study 19) 21

Supplementary Figure S8. Genomic alterations detected in PAOLA-1, OPINION, LIGHT, and Study 19... 22

References 23

Introduction

PARP inhibitor approval status

The poly(ADP-ribose) polymerase (PARP) inhibitors olaparib [1], niraparib [2], and rucaparib [3] have received global regulatory approval as first-line or second-line or later maintenance therapy in ovarian cancer. Olaparib is also approved in breast cancer, prostate cancer, and pancreatic cancer [1], rucaparib is also approved in prostate cancer [3], and the PARP inhibitor talazoparib is approved in breast cancer and prostate cancer [4].

Supplementary Table S1. Summary of ovarian cancer trials included in this analysis

Trial	Study design	Patient population	Biomarker inclusion criteria	Definition of response at study entry	Treatment (no. of patients)
SOLO1 [5-7][a]	Phase III, randomized, double-blind, multicenter (NCT01844986)	Newly diagnosed, stage III–IV, high-grade serous or high-grade endometrioid, BRCAm, CR/PR after platinum-based chemotherapy	BRCAm	**CR:** NED on imaging after chemotherapy (according to modified RECIST v1.1), and normal CA-125 level [7]<br>**PR:** ≥ 30% decrease in tumor volume from start to end of chemotherapy, or NED on imaging after chemotherapy but CA-125 level above ULN [7]	Maintenance therapy:<br>Olaparib tablets (*n* = 260) vs. placebo (*n* = 131)
PAOLA-1 [8-10]	Phase III, randomized, double-blind, multicenter (NCT02477644)	Newly diagnosed, stage III–IV, high-grade serous or high-grade endometrioid,[b] NED/CR/PR after platinum-based chemotherapy plus bevacizumab	Irrespective of biomarker status	**NED:** no measurable or assessable disease after cytoreductive surgery, plus no radiologic evidence of disease and normal CA-125 level after chemotherapy [9]<br>**CR:** disappearance of measurable or assessable disease and normalization of CA-125 levels after chemotherapy [9]<br>**PR:** radiologic evidence of disease, abnormal CA-125 level, or both [9]	Maintenance therapy:<br>Olaparib tablets + bevacizumab (*n* = 537) vs. placebo + bevacizumab (*n* = 269)

Trial	Study design	Patient population	Biomarker inclusion criteria	Definition of response at study entry	Treatment (no. of patients)
Study 19 [11-13]	Phase II, randomized, double-blind, multicenter (NCT00753545)	PSROC, high-grade serous, CR/PR after most recent platinum-based chemotherapy, ≥ 2 prior platinum-based chemotherapy regimens	Irrespective of biomarker status	**CR/PR:** Confirmed as per RECIST v1.0 and/or a CA-125 confirmed response (defined as a ≥ 50% reduction in CA-125 levels from the last pre-treatment sample, confirmed 28 days later)	Maintenance therapy: Olaparib capsules (*n* = 136) vs. placebo (*n* = 129)
SOLO2 [14, 15]	Phase III, randomized, double-blind, multicenter (NCT01874353)	PSROC, high-grade serous or high-grade endometrioid, BRCAm, CR/PR after most recent platinum-based chemotherapy, ≥ 2 prior platinum-based chemotherapy regimens	BRCAm	**CR, PR, or NED:** radiological response (modified RECIST v1.1) or NED (if optimal cytoreductive surgery was conducted prior to chemotherapy) and no evidence of rising CA-125 levels	Maintenance therapy: Olaparib tablets (*n* = 196) vs. placebo (*n* = 99)
OPINION [16, 17]	Phase IIIb, single-arm, open-label, multicenter (NCT03402841)	PSROC, high-grade serous or high-grade endometrioid, non-gBRCAm, NED/CR/PR after	Non-gBRCAm	**CR, PR, or NED:** radiological response or NED (if optimal cytoreductive surgery was conducted prior to chemotherapy) and no evidence of rising CA-125 levels	Maintenance therapy: Olaparib tablets (*n* = 279)

Trial	Study design	Patient population	Biomarker inclusion criteria	Definition of response at study entry	Treatment (no. of patients)
		most recent platinum-based chemotherapy, ≥ 2 prior platinum-based chemotherapy regimens			
LIGHT [18, 19]	Phase II, non-randomized, open-label, multicenter (NCT02983799)	PSROC, high-grade serous or high-grade endometrioid, ≥ 1 prior platinum-based chemotherapy regimens	gBRCAm; sBRCAm; HRD-positive, non-BRCAm; HRD negative[c]	N/A	Treatment<br>Olaparib tablets (*n* = 272)

Abbreviations: BRCAm, *BRCA1* and/or *BRCA2* mutation; CR, complete response; gBRCAm, germline BRCAm; HRD, homologous recombination deficiency; N/A, not applicable; NED, no evidence of disease; PR, partial response; PSROC, platinum-sensitive relapsed ovarian cancer; RECIST, Response Evaluation Criteria in Solid Tumors; sBRCAm, somatic BRCAm; ULN, upper limit of normal.

[a]Post hoc tumor HRD testing performed at Myriad.

[b]Patients with other nonmucinous epithelial ovarian cancers were eligible, provided they had a deleterious gBRCAm.

[c]Trial involved prospective, biomarker-stratified, non-comparative cohorts.

Methods

Supplementary Table S2. Assessment of mutation status and GIS in the ovarian cancer trials used in this analysis

Study	Test used for assessing gBRCAm status	Tumor tissue test used	Proportion of gBRCAm events reported in tumor tissue
SOLO1 [7]	• Myriad BRACAnalysis CDx® test	• Myriad MyChoice® CDx, PMA approved; ext. DNA	97.3% (*n* = 284/292) [20]
PAOLA-1 [9]	• Local germline test result at enrollment documented in the eCRF<br>• Where a germline test was not performed, computational assessment of germline/somatic status of tBRCAm was performed using an in-house RUO-based method implemented at Myriad	• Myriad MyChoice® HRD Plus;[a] FFPE tissue	95.5 % (*n* = 106/111)[b]
Study 19 [12, 21][c]	• Local germline test result at enrollment documented in the eCRF<br>• Where a germline test was not performed, data from central integrated BRACAnalysis® assay were used	• Myriad MyChoice® HRR deficiency test, RUO; FFPE tissue<br>• Foundation Medicine gene panel; FFPE tissue	Myriad: 96.1% (*n* = 74/77)<br>Foundation Medicine: 96.0% (*n* = 71/74) [22]
SOLO2 [15]	• Myriad BRACAnalysis CDx® test	• Myriad MyChoice® CDx, PMA approved; FFPE tissue	98.3% (*n* = 237/241) [23]
OPINION [16]	• Myriad BRACAnalysis CDx® test	• Myriad MyChoice® HRD Plus;[a] FFPE tissue	100% (*n* = 6/6)
LIGHT [18]	• Myriad BRACAnalysis CDx® test	• Myriad MyChoice® CDx, PMA approved; Myriad MyChoice® HRR deficiency test, RUO; FFPE tissue	100% (*n* = 74/74)

Abbreviations: BRCAm, *BRCA1* and/or *BRCA2* mutation; eCRF, electronic case report form; FFPE, formalin-fixed paraffin-embedded; gBRCAm, germline BRCAm; GIS, genomic instability score; HRR, homologous recombination repair; PMA, premarket approval; RUO, research use only; tBRCAm, tumor BRCAm.

[a]Now also known as MyChoice® CDx.

[b]Only measurable for a subset of patients enrolled in PAOLA-1 where local gBRCAm status was entered in the eCRF (this was not mandated in the study).

[c]In Study 19, HRR mutation status was also assessed using the Foundation Medicine gene panel (Foundation Medicine, Inc., Cambridge, MA, USA).

Supplementary Table S3. Full gene panel included in the Myriad tumor tissue test[a] (Myriad Genetic Laboratories, Inc.)

AKT1	*BRAF*	*CDKN2A*	*EPCAM*	*FGFR1*	*HOXB13*	*MAP3K1*	*NF1*	*PPP2R2A*	*RECQL*	*SMAD4*
APC	*BRCA1*	*CHEK1*	*ERBB2*	*FGFR2*	*KDM6A*	*MET*	*NFE2L2*	*PSMC3IP*	*RET*	*STAG2*
AR	*BRCA2*	*CHEK2*	*ERCC1*	*FGFR3*	*KIT*	*MLH1*	*NRAS*	*PTEN*	*RHOA*	*STK11*
ARID1A	*BRIP1*	*CSMD3*	*ERCC3*	*FGFR4*	*KLF5*	*MLH3*	*NTHL1*	*PTPRD*	*RHOB*	*TOP1*
ATM	*BTG2*	*CTCF*	*FAM175A*	*FOXA1*	*KMT2B*	*MRE11A*	*PAIP1*	*RAD51*	*RINT1*	*TP53*
ATR	*CCND3*	*DDR2*	*FANCA*	*FOXQ1*	*KMT2D*	*MSH2*	*PALB2*	*RAD51B*	*RPS20*	*TSC1*
AXIN2	*CCNE1*	*EGFR*	*FANCI*	*GALNT12*	*KRAS*	*MSH3*	*PIK3CA*	*RAD51C*	*RUNX1*	*TXNIP*
BARD1	*CDH1*	*ELF3*	*FANCL*	*GATA3*	*MAP2K1*	*MSH6*	*PMS2*	*RAD51D*	*RXRA*	*ZFP36L1*
BLM	*CDK12*	*EMSY*	*FANCM*	*GREM1*	*MAP2K2*	*MYH*	*POLD1*	*RAD54L*	*SF3B1*	
BMPR1A	*CDK4*	*EP300*	*FBXW7*	*HDAC2*	*MAP2K4*	*NBN*	*POLE*	*RB1*	*SLX4*	

[a]Research use only.

Results

Supplementary Figure S1. GIS distribution by patient race in (**A**) all patients and (**B**) patients with a tBRCAm, by tumor histology in (**C**) all patients and (**D**) patients with a tBRCAm, and by primary tumor location in (**E**) all patients and (**F**) patients with a tBRCAm

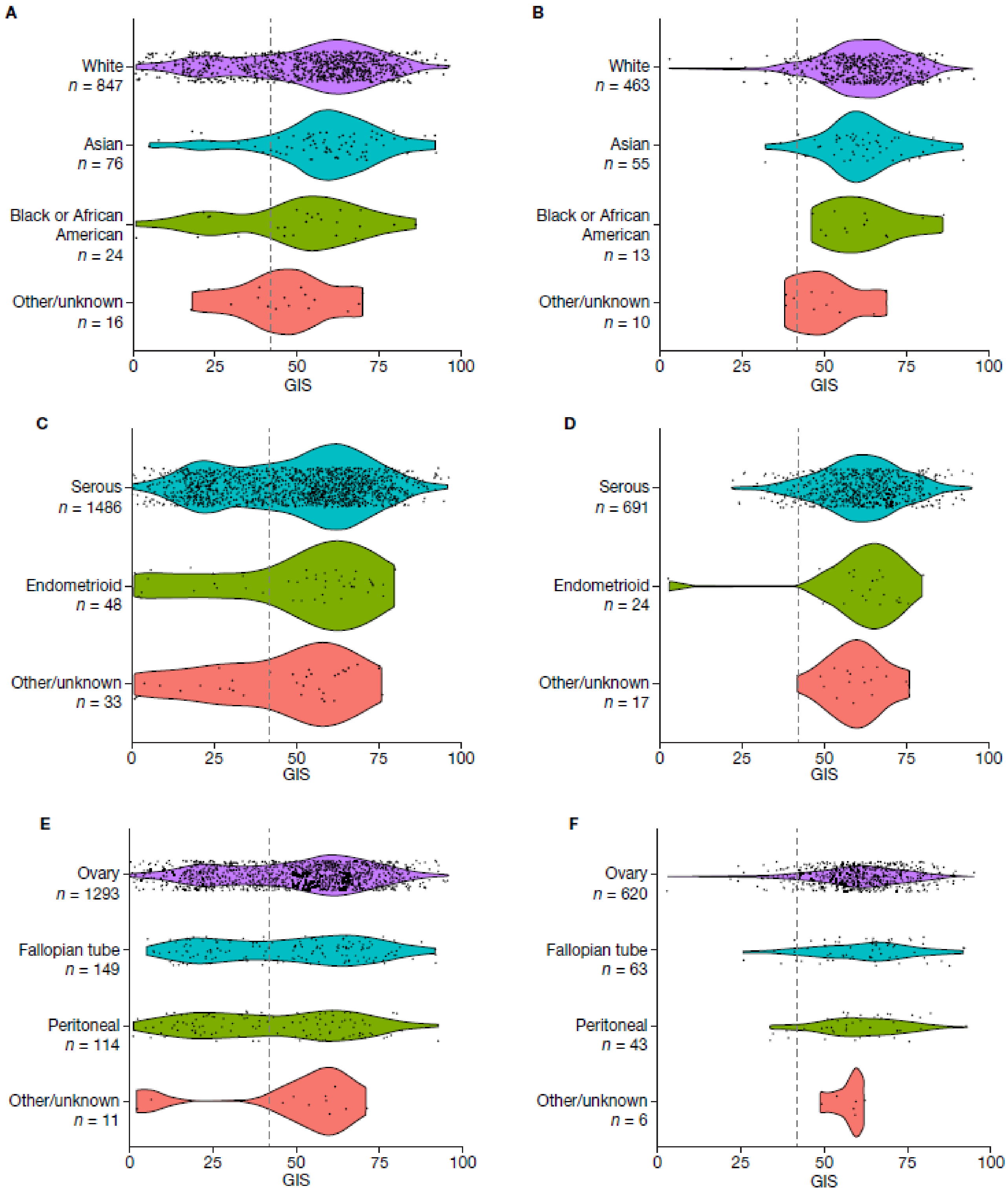

Panels **A** to **F** exclude patients who did not consent to secondary use of clinical data. Panels **A** and **B** exclude patients enrolled in PAOLA-1 as information on race was not collected in this study.
The dashed vertical lines denote the GIS cutoff of 42.
BRCAm, *BRCA1* and/or *BRCA2* mutation; GIS, genomic instability score; tBRCAm, tumor BRCAm.

Supplementary Figure S2. GIS distribution in tumors with and without tBRCAm by individual study in PAOLA-1, OPINION, LIGHT, Study 19, SOLO1, and SOLO2

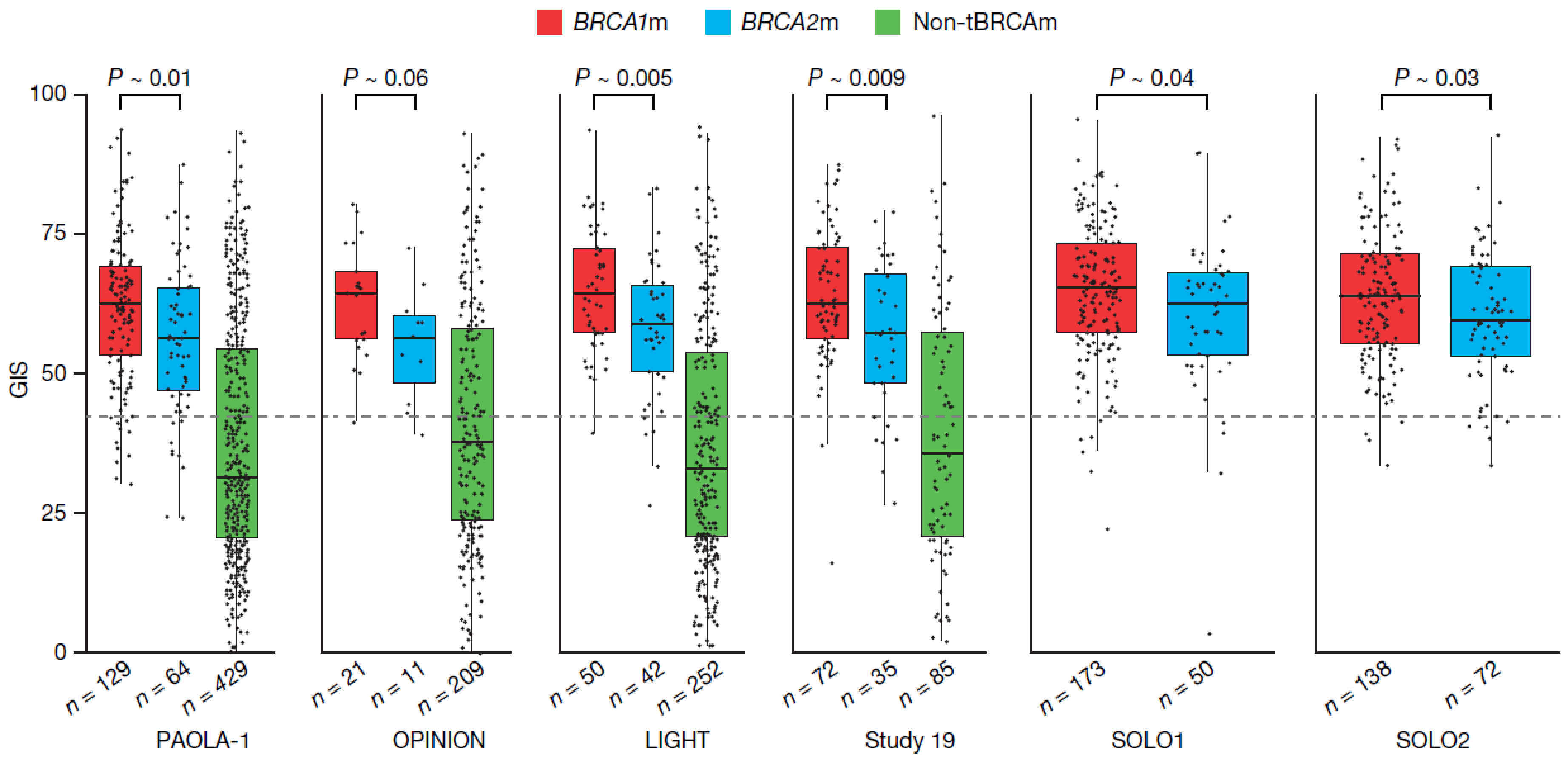

The box plot shows median (IQR) and whiskers indicate 1.5 times the IQR above Q3 and below Q1. The dashed horizontal line denotes the GIS cutoff of 42. Patients with co-occurring *BRCA1* and *BRCA2* mutations were excluded.

*BRCA1*m, *BRCA1* mutation; *BRCA2*m, *BRCA2* mutation; GIS, genomic instability score; IQR, interquartile range; Q, quartile; tBRCAm, tumor *BRCA1* and/or *BRCA2* mutation.

Supplementary Figure S3. GIS distribution in tumors from patients (**A**) in response after first-line platinum-based chemotherapy and (**B**) with platinum-sensitive relapsed disease, and (**C**) GIS distribution by germline and somatic tumor BRCAm status and in non-tBRCAm tumors

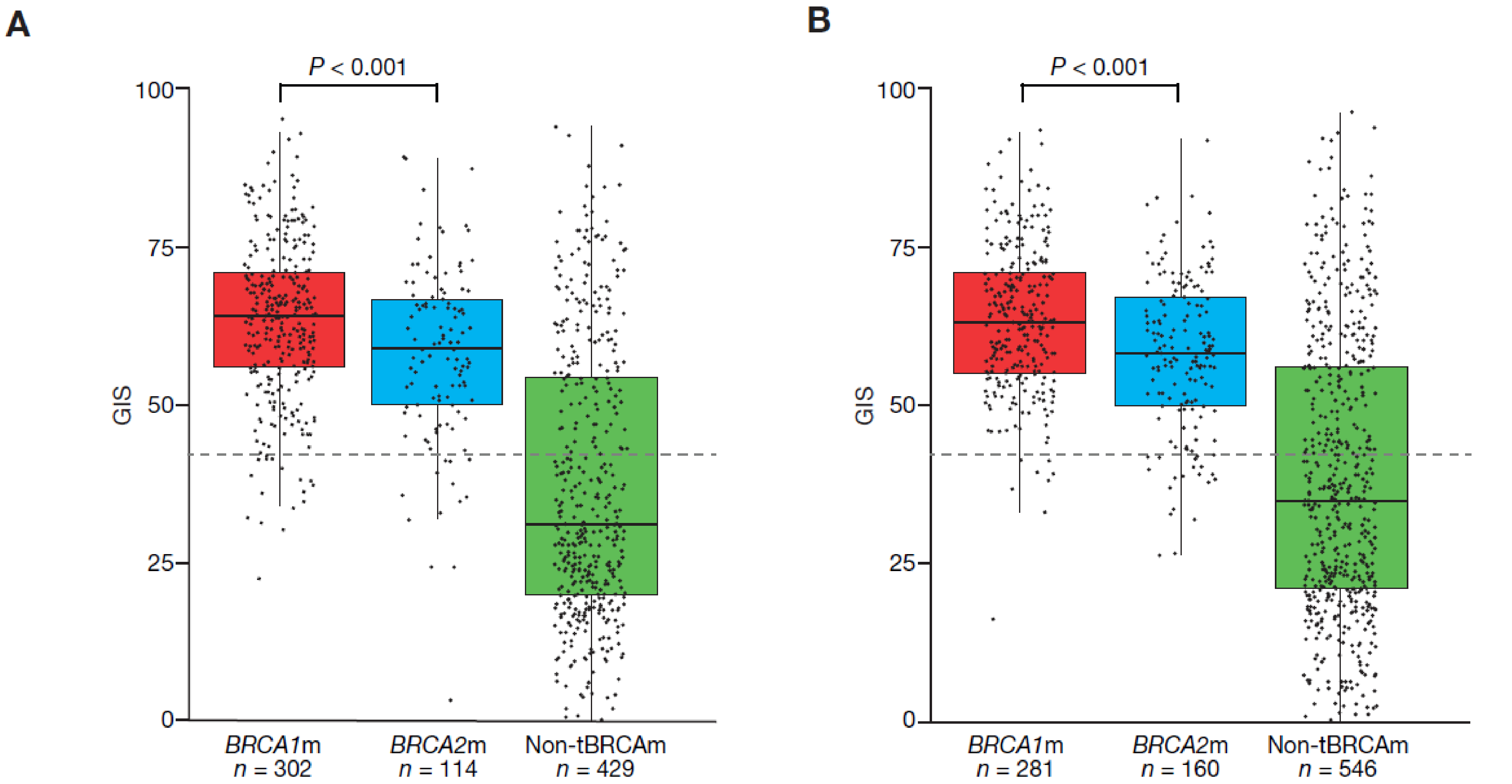

C

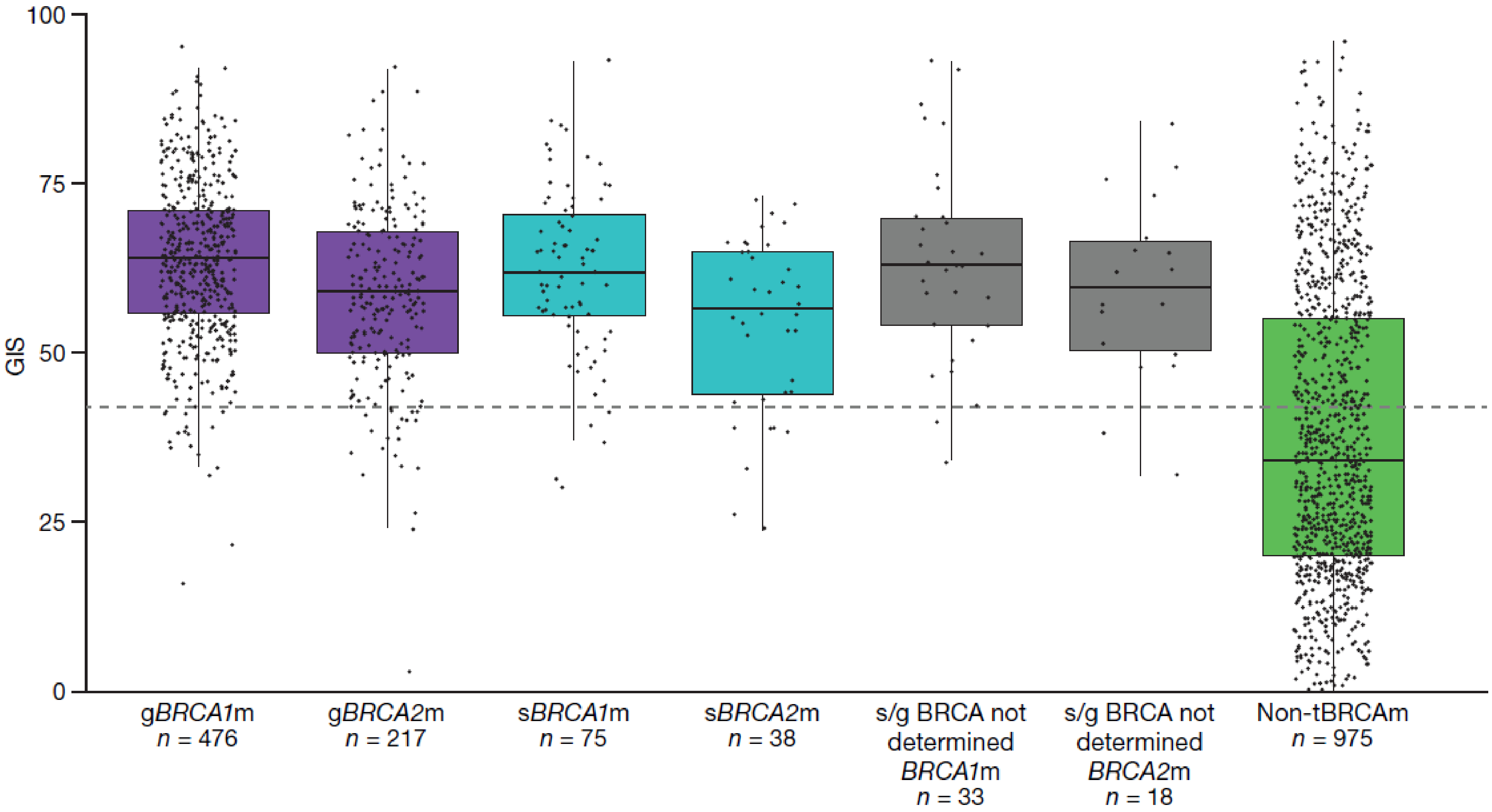

100
75
50
25
0
GIS
g*BRCA1*m
n = 476
g*BRCA2*m
n = 217
s*BRCA1*m
n = 75
s*BRCA2*m
n = 38
s/g BRCA not determined *BRCA1*m
n = 33
s/g BRCA not determined *BRCA2*m
n = 18
Non-tBRCAm
n = 975

The box plot shows median (IQR) and whiskers indicate 1.5 times the IQR above Q3 and below Q1. The dashed horizontal line denotes the GIS cutoff of 42. Excludes patients with co-occurring *BRCA1* and *BRCA2* mutations. In panel **C**, 33 tumors with *BRCA1*m and 18 tumors with *BRCA2*m could not be classified as germline or somatic.

*BRCA1*m, *BRCA1* mutation; *BRCA2*m, *BRCA2* mutation; g*BRCA1*m, germline *BRCA1* mutation; g*BRCA2*m, germline *BRCA2* mutation; GIS, genomic instability score; IQR, interquartile range; Q, quartile; s*BRCA1*m, somatic BRCA*1* mutation; s*BRCA2*m, somatic *BRCA2* mutation; s/g, somatic/germline; tBRCAm, tumor *BRCA1* and/or *BRCA2* mutation.

Supplementary Figure S4. (**A**) Gene-specific zygosity in tumors with *BRCA1*m or *BRCA2*m, (**B**) gene-specific zygosity and the rate of biallelic loss in tumors with a BRCAm, and (**C**) gene-specific zygosity and the rate of biallelic loss in tumors with germline *BRCA1*m or *BRCA2*m and somatic *BRCA1*m or *BRCA2*m

A

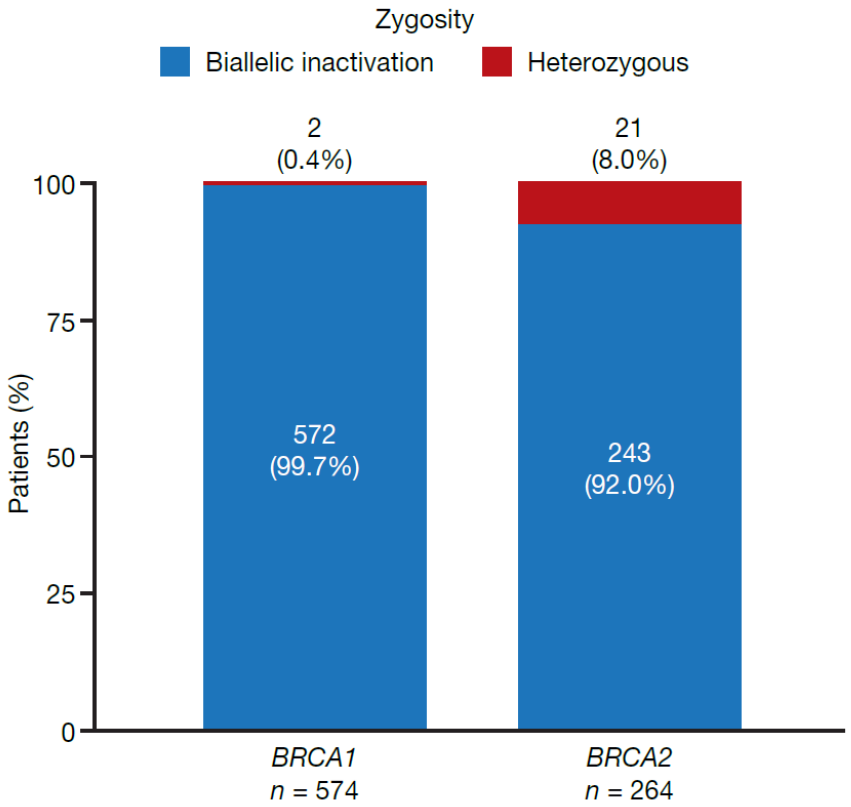

B

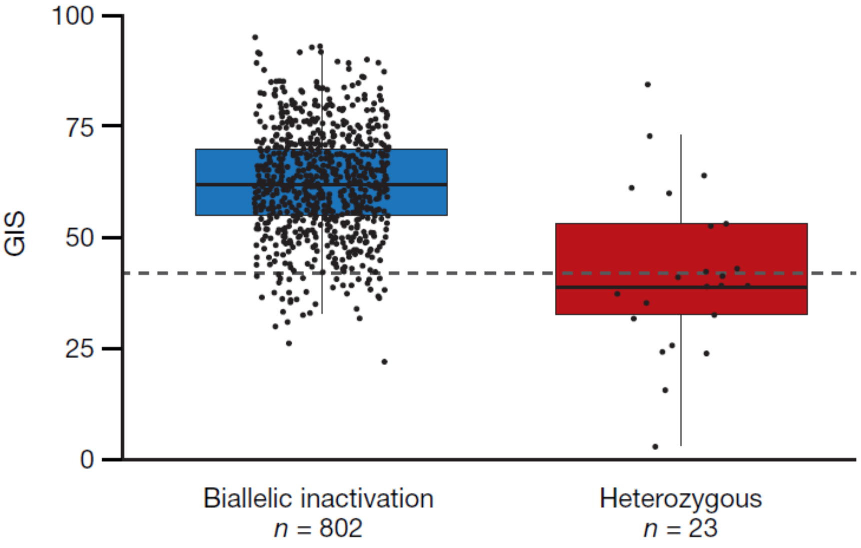

C

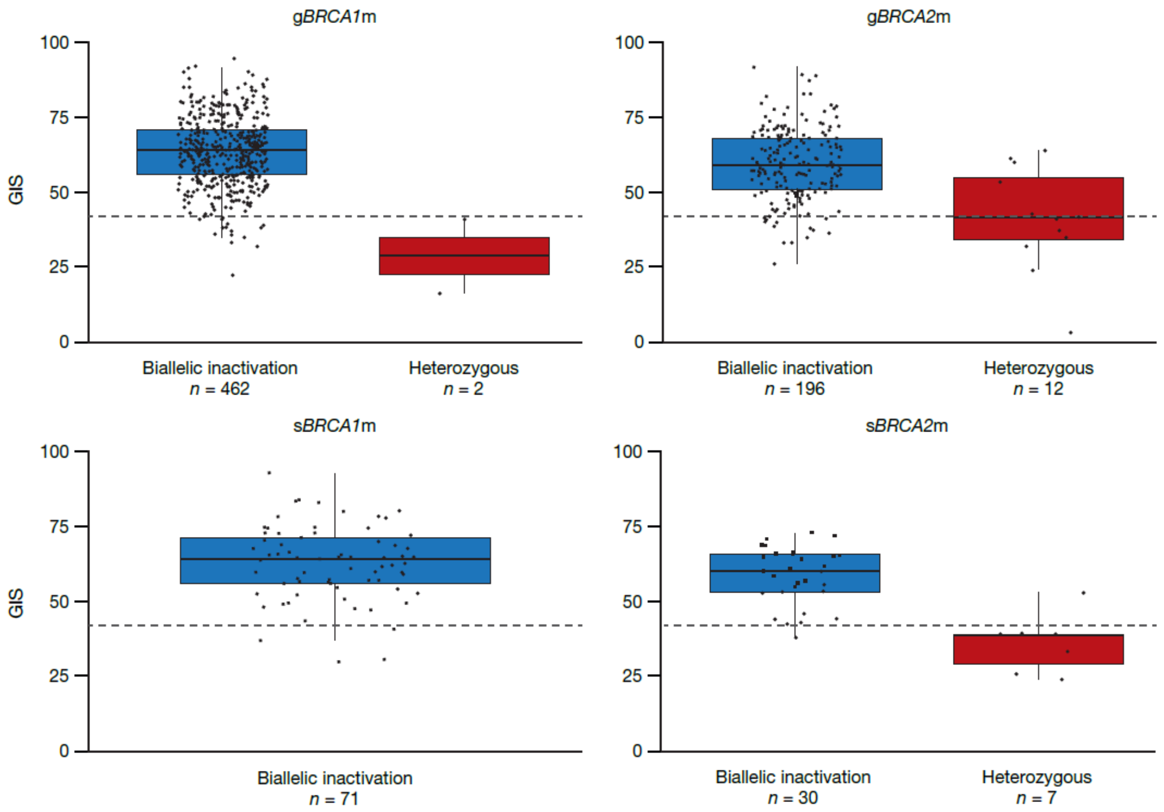

Panel **A** shows results for 838 patients with a tBRCAm and evaluable zygosity. Excludes patients with co-occurring *BRCA1* and *BRCA2* mutations as gene-specific zygosity on the patient level cannot be assessed in these cases. Cases where gene-specific zygosity could not be assessed are not shown. In panels **B** and **C**, the box plot shows median (IQR) and whiskers indicate 1.5 times the IQR above Q3 and below Q1. The dashed horizontal line denotes the GIS cutoff of 42. In panel **B**, results are shown for 825 BRCAm tumors with evaluable zygosity. In panel **C**, results are shown for 672 gBRCAm tumors and 108 somatic BRCAm tumors with evaluable zygosity; heterozygosity could not be classified in 21 gBRCAm tumors and 5 somatic BRCAm tumors.

*BRCA1*m, *BRCA1* mutation; *BRCA2*m, *BRCA2* mutation; BRCAm, *BRCA1* and/or *BRCA2* mutation; g*BRCA1*m, germline *BRCA1* mutation; g*BRCA2*m, germline *BRCA2* mutation; gBRCAm, germline BRCA mutation; GIS, genomic instability score; IQR, interquartile range; Q, quartile; s*BRCA1*m, somatic *BRCA1* mutation; s*BRCA2*m, somatic *BRCA2* mutation; tBRCAm, tumor BRCAm.

Supplementary Figure S5. Gene-specific zygosity in patients with *BRCA1*m or *BRCA2*m by individual study

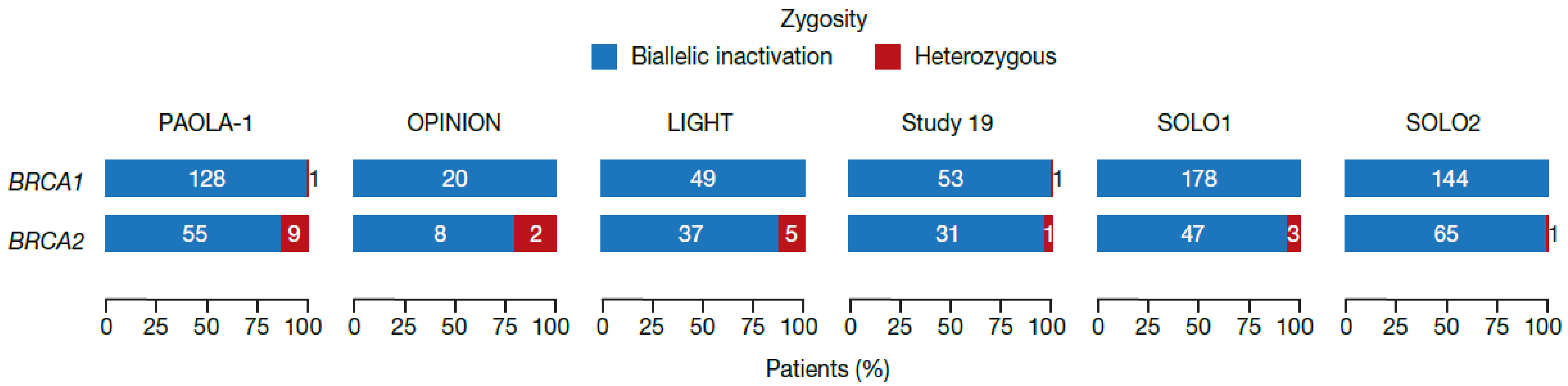

Excludes patients with co-occurring *BRCA1* and *BRCA2* mutations, as gene-specific zygosity on the patient level cannot be assessed in these cases. Cases where gene-specific zygosity could not be assessed are not shown.

*BRCA1*m, *BRCA1* mutation; *BRCA2*m, *BRCA2* mutation.

Supplementary Figure S6. GIS distribution in (**A**) non-BRCA HRRm tumors from patients in response after first-line platinum-based chemotherapy and with platinum-sensitive relapsed disease and (**B**) in tumors with non-BRCA HRRm by individual study in PAOLA-1, OPINION, LIGHT, and Study 19

A

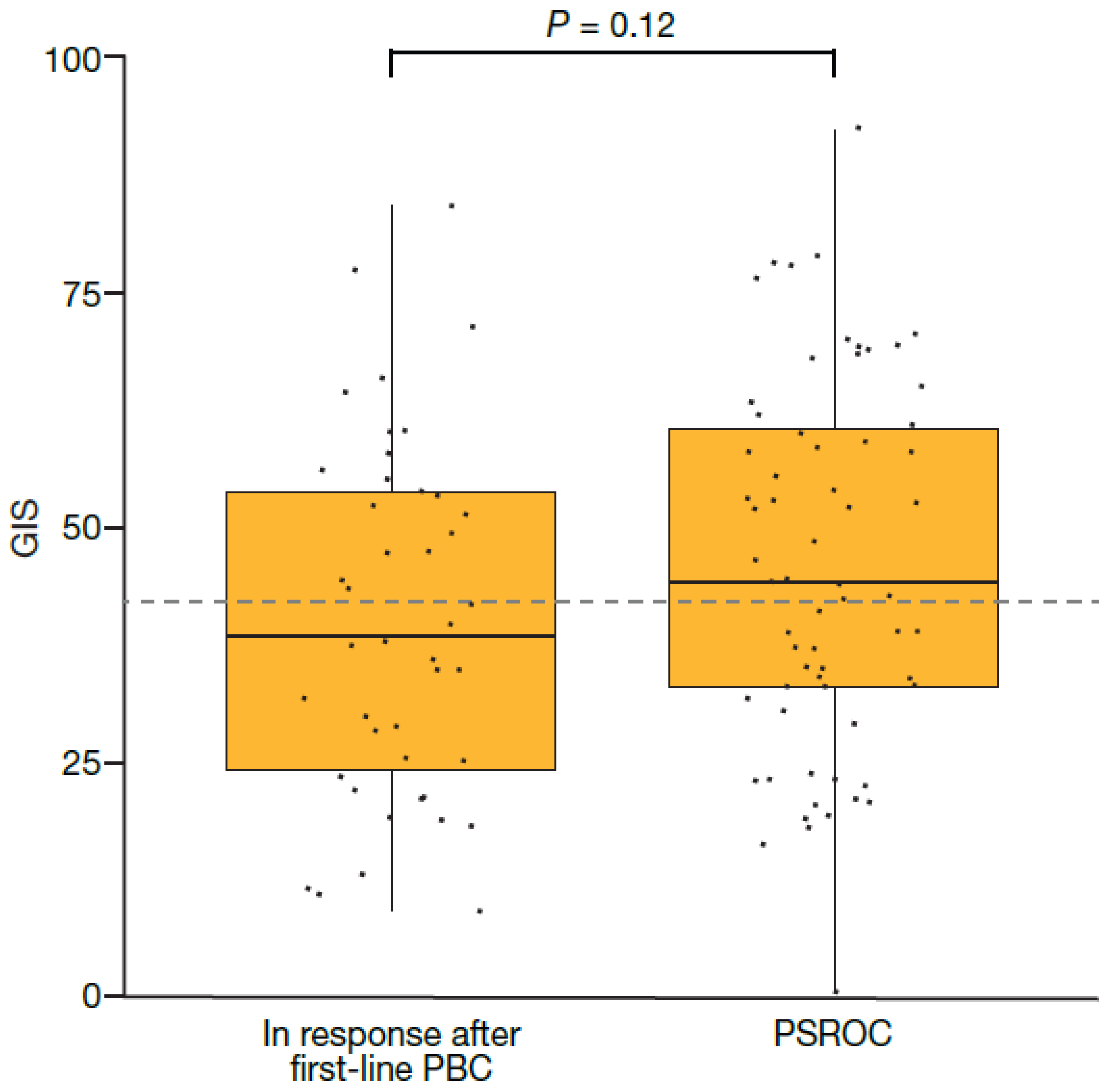

B

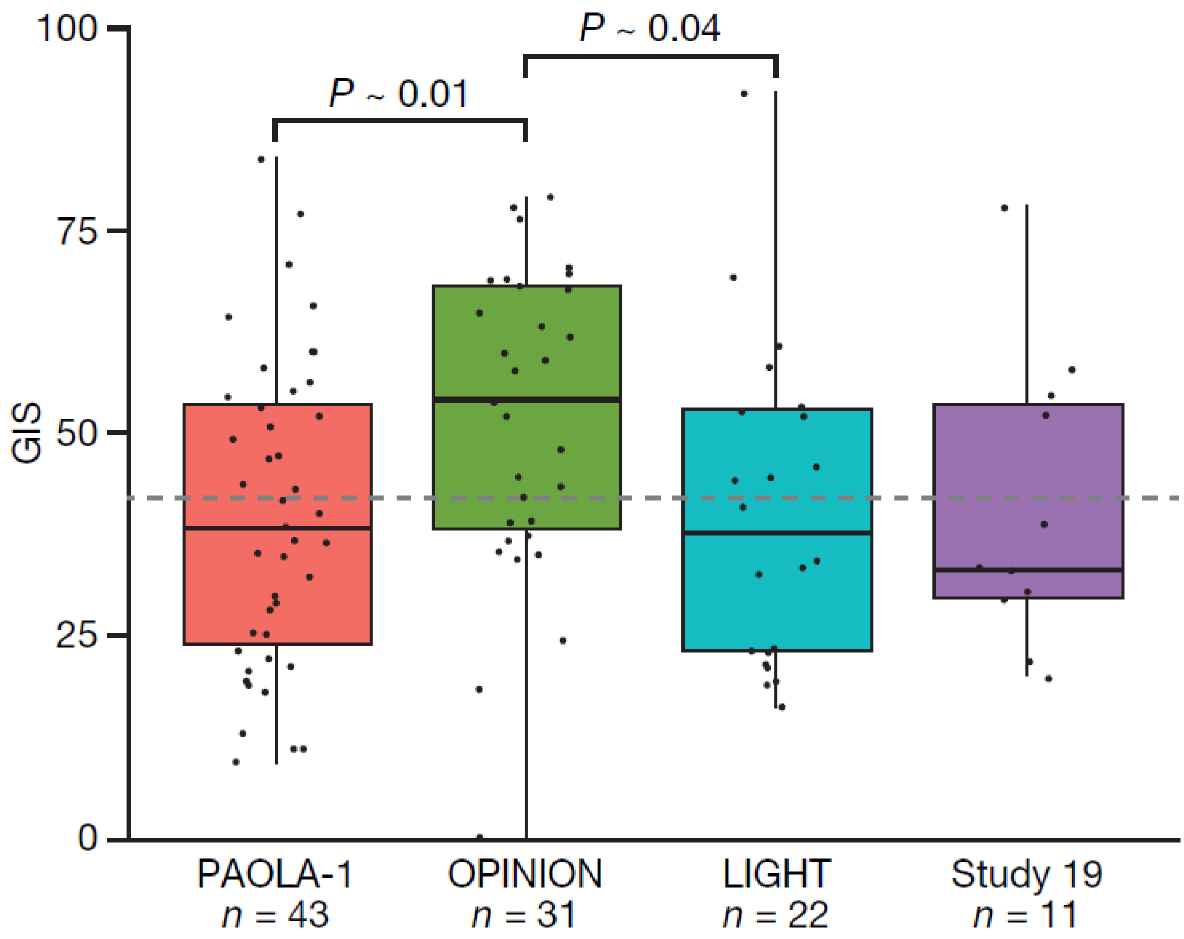

The box plot shows median (IQR) and whiskers indicate 1.5 times the IQR above Q3 and below Q1. The dashed horizontal line denotes the GIS cutoff of 42.

BRCA, *BRCA1* and/or *BRCA2*; GIS, genomic instability score; HRRm, homologous recombination repair mutation; IQR, interquartile range; PBC, platinum-based chemotherapy; PSROC, platinum-sensitive relapsed ovarian cancer; Q, quartile.

Supplementary Figure S7. GIS distribution in patients with non-BRCA HRRm by individual study (PAOLA-1, OPINION, LIGHT, and Study 19)

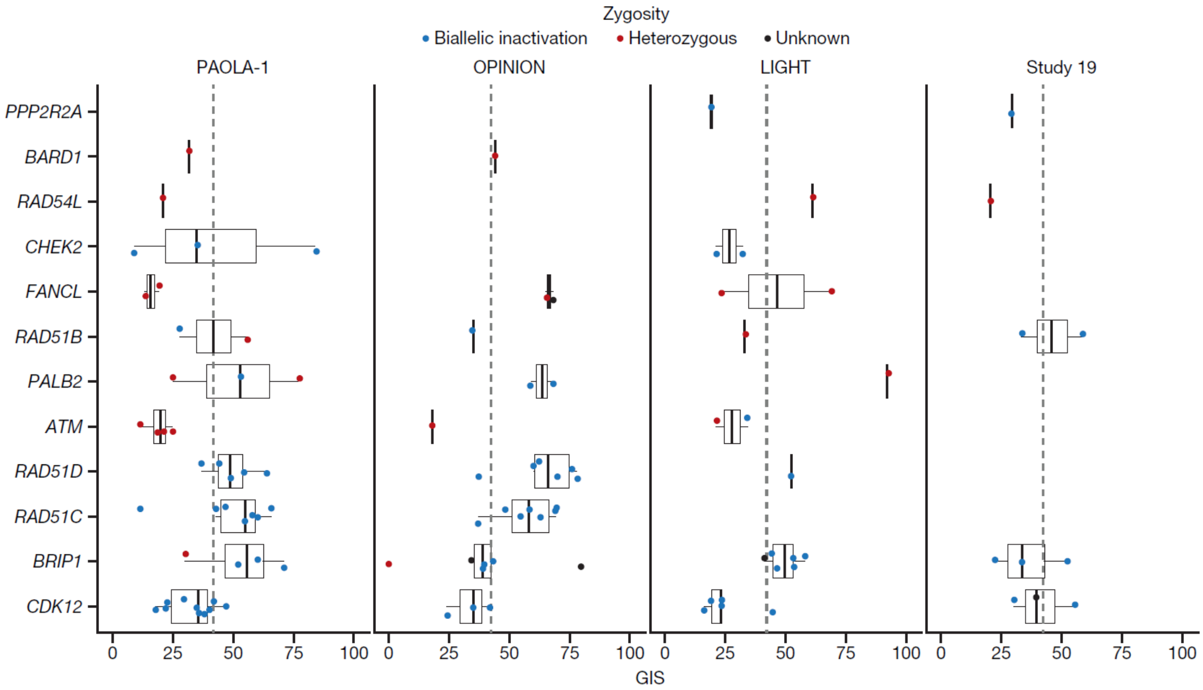

The box plot shows median (IQR) and whiskers indicate 1.5 times the IQR above Q3 and below Q1. The dashed horizontal line denotes the GIS cutoff of 42.

Excludes tumors with co-occurring HRR, as gene-specific HRD and zygosity on the patient level cannot be assessed, and only includes 103 patient samples where a GIS was calculated. An additional gene (*CHEK1*) is not shown, as no individual mutations were detected.

BRCA, *BRCA1* and/or *BRCA2*; GIS, genomic instability score; HRD, homologous recombination deficiency; HRR, homologous recombination repair; HRRm, HRR mutation; IQR, interquartile range; Q, quartile.

Supplementary Figure S8. Genomic alterations detected in PAOLA-1, OPINION, LIGHT, and Study 19

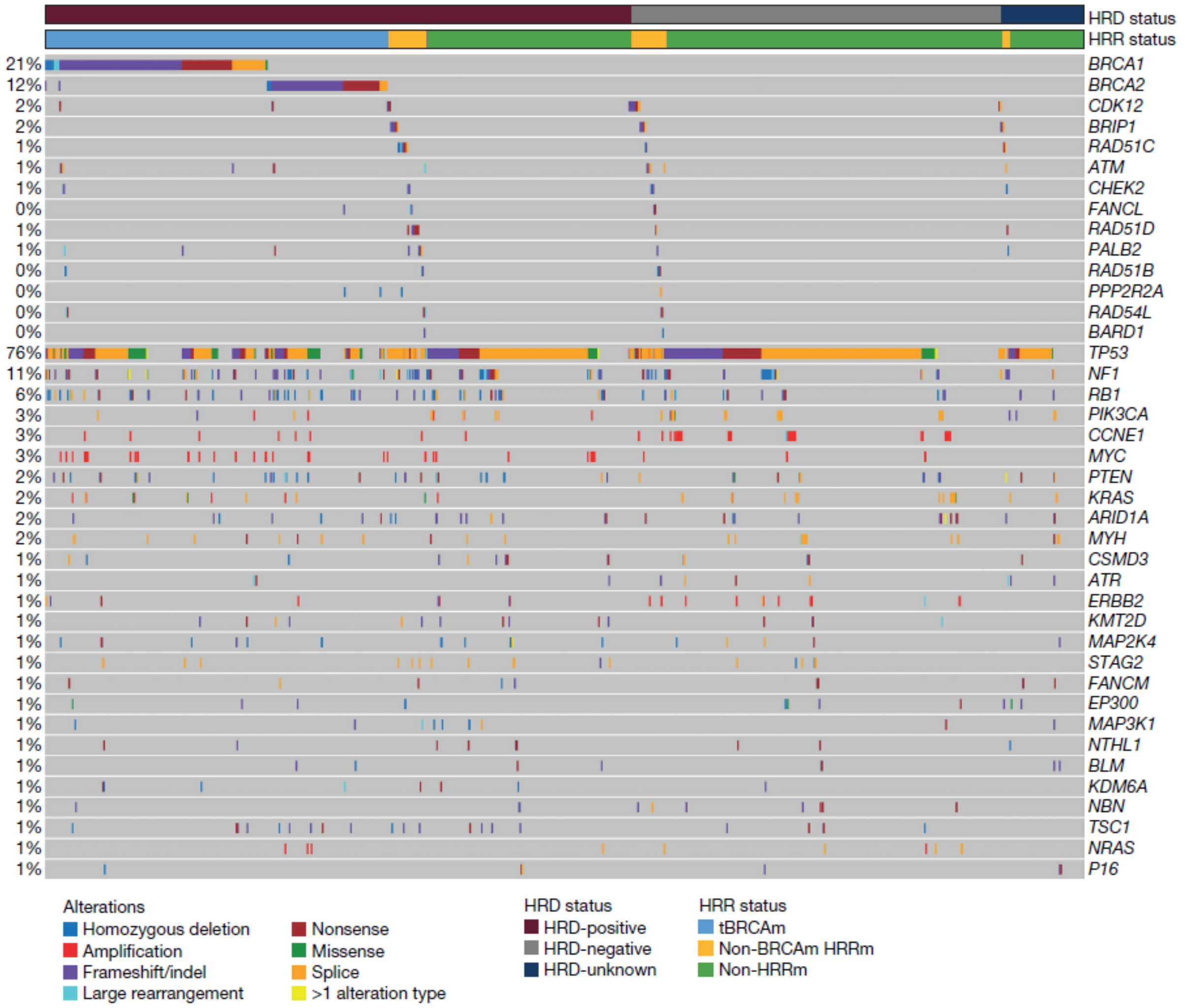

BRCAm, *BRCA1* and/or *BRCA2* mutation; HRD, homologous recombination deficiency; HRR, homologous recombination repair; tBRCAm, tumor BRCAm.

References

1. AstraZeneca. LYNPARZA® (olaparib) tablets, for oral use: prescribing information 2023. https://www.accessdata.fda.gov/drugsatfda_docs/label/2023/208558s029lbl.pdf. Accessed 30 Oct 2023.
2. GlaxoSmithKline. ZEJULA® (niraparib) capsules, for oral use: prescribing information 2023. https://www.accessdata.fda.gov/drugsatfda_docs/label/2023/208447s027lbl.pdf. Accessed 4 Aug 2023.
3. Clovis Oncology. RUBRACA® (rucaparib) tablets, for oral use: prescribing information 2022. https://www.accessdata.fda.gov/drugsatfda_docs/label/2022/209115s013lbl.pdf. Accessed 4 Aug 2023.
4. Pfizer. TALZENNA® (talazoparib) capsules, for oral use: prescribing information 2023. https://www.accessdata.fda.gov/drugsatfda_docs/label/2023/211651s010lbl.pdf. Accessed 4 Aug 2023.
5. Banerjee S, Moore KN, Colombo N, Scambia G, Kim BG, Oaknin A, et al. Maintenance olaparib for patients with newly diagnosed advanced ovarian cancer and a BRCA mutation (SOLO1/GOG 3004): 5-year follow-up of a randomised, double-blind, placebo-controlled, phase 3 trial. Lancet Oncol. 2021;22(12):1721-31.
6. DiSilvestro P, Banerjee S, Colombo N, Scambia G, Kim BG, Oaknin A, et al. Overall survival with maintenance olaparib at a 7-year follow-up in patients with newly diagnosed advanced ovarian cancer and a BRCA mutation: the SOLO1/GOG 3004 trial. J Clin Oncol. 2023;41(3):609-17.
7. Moore K, Colombo N, Scambia G, Kim B-G, Oaknin A, Friedlander M, et al. Maintenance olaparib in patients with newly diagnosed advanced ovarian cancer. N Engl J Med. 2018;379(26):2495-505.
8. González-Martín A, Desauw C, Heitz F, Cropet C, Gargiulo P, Berger R, et al. Maintenance olaparib plus bevacizumab in patients with newly diagnosed advanced high-grade ovarian cancer: main analysis of second progression-free survival in the phase III PAOLA-1/ENGOT-ov25 trial. Eur J Cancer. 2022;174:221-31.
9. Ray-Coquard I, Pautier P, Pignata S, Pérol D, González-Martín A, Berger R, et al. Olaparib plus bevacizumab as first-line maintenance in ovarian cancer. N Engl J Med. 2019;381(25):2416-28.
10. Ray-Coquard I, Leary A, Pignata S, Cropet C, Gonzalez-Martin A, Marth C, et al. Olaparib plus bevacizumab first-line maintenance in ovarian cancer: final overall survival results from the PAOLA-1/ENGOT-ov25 trial. Ann Oncol. 2023;34(8):681-92.
11. Friedlander M, Matulonis U, Gourley C, du Bois A, Vergote I, Rustin G, et al. Long-term efficacy, tolerability and overall survival in patients with platinum-sensitive, recurrent high-grade serous ovarian cancer treated with maintenance olaparib capsules following response to chemotherapy. Br J Cancer. 2018;119(9):1075-85.
12. Ledermann J, Harter P, Gourley C, Friedlander M, Vergote I, Rustin G, et al. Olaparib maintenance therapy in platinum-sensitive relapsed ovarian cancer. N Engl J Med. 2012;366(15):1382-92.
13. Ledermann J, Harter P, Gourley C, Friedlander M, Vergote I, Rustin G, et al. Olaparib maintenance therapy in patients with platinum-sensitive relapsed serous ovarian cancer: a preplanned retrospective analysis of outcomes by BRCA status in a randomised phase 2 trial. Lancet Oncol. 2014;15(8):852-61.
14. Poveda A, Floquet A, Ledermann JA, Asher R, Penson RT, Oza AM, et al. Olaparib tablets as maintenance therapy in patients with platinum-sensitive relapsed ovarian cancer and a BRCA1/2 mutation (SOLO2/ENGOT-Ov21): a final analysis of a double-blind, randomised, placebo-controlled, phase 3 trial. Lancet Oncol. 2021;22(5):620-31.
15. Pujade-Lauraine E, Ledermann JA, Selle F, Gebski V, Penson RT, Oza AM, et al. Olaparib tablets as maintenance therapy in patients with platinum-sensitive, relapsed ovarian cancer and a BRCA1/2 mutation (SOLO2/ENGOT-Ov21): a double-blind, randomised, placebo-controlled, phase 3 trial. Lancet Oncol. 2017;18(9):1274-84.

16. Poveda A, Lheureux S, Colombo N, Cibula D, Lindemann K, Weberpals J, et al. Olaparib maintenance monotherapy in platinum-sensitive relapsed ovarian cancer patients without a germline BRCA1/BRCA2 mutation: OPINION primary analysis. Gynecol Oncol. 2022;164(3):498-504.
17. Poveda A, Lheureux S, Colombo N, Cibula D, Elstrand M, Weberpals J, et al. 531P Maintenance olaparib monotherapy in patients (pts) with platinum-sensitive relapsed ovarian cancer (PSR OC) without a germline BRCA1/BRCA2 mutation (non-gBRCAm): final overall survival (OS) results from the OPINION trial. Ann Oncol. 2022;33:S790.
18. Cadoo K, Simpkins F, Mathews C, Liu YL, Provencher D, McCormick C, et al. Olaparib treatment for platinum-sensitive relapsed ovarian cancer by BRCA mutation and homologous recombination deficiency status: Phase II LIGHT study primary analysis. Gynecol Oncol. 2022;166(3):425-31.
19. Mathews CA, Simpkins F, Cadoo KA, Liu YL, Provencher DM, McCormick C, et al. Olaparib treatment (Tx) in patients (pts) with platinum-sensitive relapsed ovarian cancer (PSR OC) by BRCA mutation (BRCAm) and homologous recombination deficiency (HRD) status: overall survival (OS) results from the phase II LIGHT study. J Clin Oncol. 2021;39(15_suppl):5515.
20. Myriad Genetic Laboratories. MyChoice® CDx technical information. https://myriad-web.s3.amazonaws.com/myChoiceCDx/downloads/myChoiceCDxTech.pdf. Accessed 11 Mar 2024.
21. Hodgson DR, Dougherty BA, Lai Z, Fielding A, Grinsted L, Spencer S, et al. Candidate biomarkers of PARP inhibitor sensitivity in ovarian cancer beyond the BRCA genes. Br J Cancer. 2018;119(11):1401-9.
22. Dougherty BA, Lai Z, Hodgson DR, Orr MCM, Hawryluk M, Sun J, et al. Biological and clinical evidence for somatic mutations in BRCA1 and BRCA2 as predictive markers for olaparib response in high-grade serous ovarian cancers in the maintenance setting. Oncotarget. 2017;8(27):43653-61.
23. Hodgson DR, Brown JS, Dearden SP, Lai Z, Elks CE, Milenkova T, et al. Concordance of BRCA mutation detection in tumor versus blood, and frequency of bi-allelic loss of BRCA in tumors from patients in the phase III SOLO2 trial. Gynecol Oncol. 2021;163(3):563-8.
